# Effect of Photoperiod on Growth and Production of Trichothecene Mycotoxins in *Fusarium graminearum*

**DOI:** 10.3390/toxins18100459

**Published:** 2026-10-21

**Authors:** William T. Hay, Susan P. McCormick, Nathan D. Kemp, Mark Busman, Daren W. Brown, Hye-Seon Kim, Carson M. Andorf, Stephen F. Harding, Robert H. Proctor, Martha M. Vaughan

**Affiliations:** 1 USDA, Mycotoxin Prevention and Applied Microbiology Unit, Agricultural Research Service, National Center for Agricultural Utilization Research, 1815 N. University Street, Peoria, IL 61604, USA; susan.mccormick@usda.gov (S.P.M.); nathan.kemp@usda.gov (N.D.K.); mark.busman@usda.gov (M.B.); daren.brown@usda.gov (D.W.B.); hyeseon.kim@usda.gov (H.-S.K.); robert.proctor@usda.gov (R.H.P.); martha.vaughan@usda.gov (M.M.V.); 2 USDA, Agricultural Research Service, Corn Insects and Crop Genetics Research Unit, Iowa State University, Ames, IA 50011, USA; carson.andorf@usda.gov (C.M.A.); stephen.harding@usda.gov (S.F.H.)

**Keywords:** *Fusarium*, photoperiod, trichothecenes, transcriptomics, mycotoxins, corn, wheat, food safety

## Abstract

*Fusarium graminearum* is a plant-pathogenic and mycotoxin-producing fungus of major concern to cereal production worldwide. There are genetically distinct populations of the pathogen that are associated with different trichothecene mycotoxin profiles. A recent study has identified key differences in genes associated with light response and circadian regulation among the major North American *F. graminearum* populations. It is currently unclear how these populations differ in their response to photoperiod (hours of light per day). Therefore, we evaluated the effects of photoperiod on growth and trichothecene production in isolates representing the three most prominent North American populations (NA1, NA2 and NA3) of *F. graminearum*. Photoperiod had a profound effect on fungal growth, colony morphology, and trichothecene production. This effect varied by substrate (i.e., fungal growth medium or plant host) and among pathogen populations. Photoperiod had a considerable impact on inoculated corn seedlings, and total trichothecene production was substantially greater (2–25×) in inoculated corn compared with wheat. Photoperiod had little effect on trichothecene production in inoculated wheat seedlings, except for NA3. Differences in trichothecene production were not due to differences in fungal biomass, but likely the result of photoperiod-dependent gene expression changes to trichothecene biosynthesis.

## 1. Introduction

*Fusarium* species are responsible for some of the most devastating diseases of cereal crops worldwide [1,2]. These diseases reduce yield and grain quality and result in contamination of grain with mycotoxins, such as trichothecenes, that pose serious health hazards to humans and animals [3,4]. *Fusarium*-incited crop diseases and mycotoxin contamination are heavily influenced by weather and other environmental factors [5,6]. Trichothecene mycotoxins are formed from the primary metabolite farnesyl pyrophosphate via a complex biosynthetic pathway and vary widely in structure and toxicity [7,8,9]. Multiple trichothecenes, such as deoxynivalenol (DON), are acutely phytotoxic, and cause rapid plant cell death. In wheat, DON is considered a virulence factor that enhances the ability of *Fusarium graminearum* to cause *Fusarium* head blight (FHB) [10,11,12]. In mammals, DON can cause vomiting, liver damage, immune suppression, and an increased risk of secondary bacterial infection [13,14,15,16].

In North America, the species *F. graminearum* causes FHB, and genetically distinct populations are often categorized by their trichothecene production profile, otherwise referred to as chemotype [17]. The three primary chemotypes for *F. graminearum* are 15-acetyl-deoxynivalenol (15-ADON), 3-acetyl-deoxynivalenol (3-ADON), and 7α-hydroxy, 15-deacetylcalonectrin (NX-2), which are associated with three genetically distinct North American populations of *F. graminearum*: NA1, NA2, and NA3, respectively [18,19]. During plant infection, 15-ADON and 3-ADON are typically deacetylated into DON, while NX-2 is deacetylated into 7α-hydroxy,3,15-dideacetylcalonectrin (NX-3) [20,21]. Collectively, NX-2 and NX-32 have been called NX toxins. These populations have different geographic distributions: NA1 is the predominant population throughout North America and can be found from Louisiana to northeastern Canada, NA2 in the midwestern U.S. and the prairie and maritime provinces of Canada, and NA3 in southern Canada and the northern U.S. [18,22,23]. Environmental factors play an important role in disease outcomes, and *F. graminearum* populations are geographically distributed in areas with varying photoperiod during the wheat growing season. For example, more northern wheat production regions in Canada experience longer day lengths (16–17 h) during their growing season compared with production regions in the southern U.S. (12–13 h) [24]. Additionally, a recent study has identified key differences in genes associated with light response and circadian regulation among *F. graminearum* populations [18]. An ortholog of the circadian clock gene *frequency* (*frq*) occurred in only the NA1 population, and a gene related to the cryptochrome CryD, a blue/UV-A photoreceptor typically associated with light response and circadian regulation, was found to be rapidly increasing in frequency in NA3. It currently remains unclear to what extent photoperiod affects trichothecene production in *F. graminearum* and whether any effects vary by population.

Light has a profound effect on fungal biology and is used to set a circadian clock that regulates growth, development, and reproduction [25]. Fungi can form highly complex and intricate symbiotic relationships with plants, exchanging metabolites, bolstering defenses and altering development [26,27]. Pathogenic fungi frequently employ similarly complex tactics to exploit plant hosts. These plant–fungus interactions are often significantly influenced by photoperiod, the number of hours of light in a day [28]. The circadian flux of plant metabolites influences the abundance, diversity, and behavior of microorganisms intimately associated with the plants [29,30,31].

The white-collar complex (WCC) is the primary regulator of light response in ascomycetous fungi. The most thorough analyses of the WCC have been done in *Neurospora crassa*, but more limited analyses of *Fusarium fujikuroi*, *F. graminearum* and *F. oxysporum* suggest that WCC operates similarly in *Fusarium* [32,33,34,35,36]. WCC acts directly on light-regulated genes and by regulating other photoreceptor genes [37]. WCC is considered the most significant photoreceptor in fungi, and while there are other photoreceptors, they typically have minor secondary functions [38]. The WCC is composed of a light–oxygen–voltage domain (LOV) that binds flavin and absorbs blue light (WC-1) and a zinc finger DNA-binding domain (WC-2) [39,40]. Together, they form a blue-light activated heterodimer that functions as a transcription factor. WCC also controls and modulates photoinduction of the proteins cryptochrome CryD (blue), flavoprotein Vvda (blue), rhodopsin CarO (green) and photolyase PhrA (blue) [32,41,42]. However, WCC acts more as a master regulatory protein by altering, activating, or repressing secondary metabolism independent of light, often to a greater degree than that due to light induction [37,43]. The WCC also has a role in carotenogenesis, UV resistance, hydrophobicity, and virulence in *Fusarium* [35]. However, the role of the WCC in trichothecene biosynthesis is complicated, as it functions as both a light-dependent and light-independent regulator [33].

In *F. graminearum*, light stimulates conidiation and sexual development, including the formation of fruiting bodies (perithecia) and the release of sexual spores (ascospores) [44,45,46]. The full spectrum of light, including near-UV light (UV-A), is important for promoting sporulation and perithecia formation [47]. Light can also cause significant stress on fungi, principally due to UV radiative damage and the production of reactive oxygen species, leading to the biosynthesis of defensive secondary metabolites and the accumulation of protective pigments [33,44].

The nutrient composition of a host or growth medium also strongly influences the production of secondary metabolites such as trichothecenes. Trichothecene production is strongly induced by sucrose or simple sugars such as 1-kestose, nystose, or raffinose, which contain sucrose moieties [48,49]. However, sucrose is not directly involved in the trichothecene biosynthesis. Similarly, nitrogenous polyamines such as agmatine, putrescine, arginine, and guanine can induce trichothecene production but are not substrates or cofactors in trichothecene biosynthesis [50]. A minimal medium formulation that includes sucrose and agmatine sulfate as the sole source of nitrogen will strongly induce trichothecene biosynthesis in *F. graminearum* and can be used as a basis to compare the effects of abiotic factors on trichothecene production [51].

In the current literature, it is unclear how photoperiod affects trichothecene production in *F. graminearum* and whether any effects vary among North American populations of the fungus. Therefore, we evaluated how photoperiod influences trichothecene production across the NA1, NA2, and NA3 populations of *F. graminearum* and whether these effects differed among the populations and/or substrates. We also assessed differential gene expression in *F. graminearum* in response to photoperiod. This study provides evidence for the influence of photoperiod on trichothecene production among the three *F. graminearum* populations and for how the effects differ by substrate (i.e., fungal growth medium or plant host). The study also identified photoperiod-dependent changes in the expression of *F. graminearum* genes related to light response, pathogenicity, and secondary metabolite production.

## 2. Results

### 2.1. Growth Medium, Photoperiod and Colony Morphology

**Figure 1:**
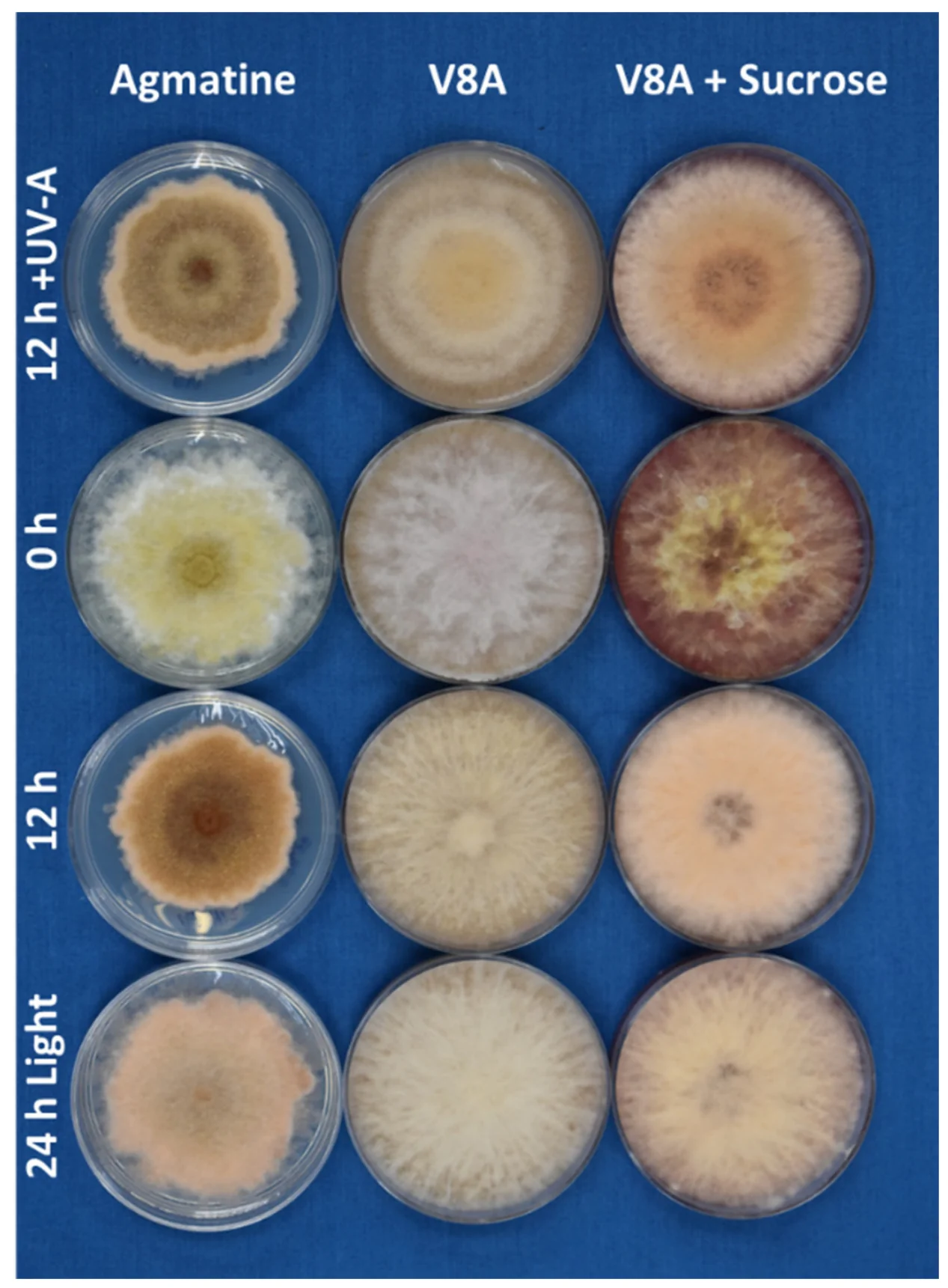
Effect of photoperiod on *Fusarium graminearum* Gz3639 on three agar media (agmatine, V8, and V8 supplemented with sucrose) after seven days of growth. Light conditions were either 0, 12, or 24 h/day of fluorescent light (60 µmol m^−2^ s^−1^) or a 12 h fluorescent light/day supplemented with UV-A.

Altering photoperiod (hours of light per day during fungal growth) caused changes in colony morphology, spread, and pigmentation of strain Gz3639, a member of the NA1 population of *F. graminearum*. The fungus was grown on three agar media: agmatine, V8, and V8 supplemented with sucrose (Figure 1). Although each medium resulted in a unique colony morphology, morphologies on the two V8 media were more similar to one another than either was to the morphology on agmatine medium. Each light condition, 0, 12, and 24 h light/day induced differences in colony morphology, including pigmentation, growth rate, and density of mycelia. The 0 h light/day treatment induced the fastest radial growth, but the resulting colonies lacked the red pigmentation characteristic of aurofusarin [52].

The impact of photoperiod differed on the agmatine medium versus the two V8 media. With at least 12 h light/day, *F. graminearum* colonies consisted of dense and relatively slow-growing mycelia on agmatine medium but less dense and faster-growing on the V8 media. However, photoperiod affected mycelial density on the media: longer light duration induced thicker mycelial mats, a continuous sheet that was nearly leather-like, with greater reddish-brown and orange-tan pigment deposits suggestive of aurofusarin or rubrofusarin [52]. GC-MS analysis of trichothecene content in the cultures revealed that *F. graminearum* produced the mycotoxins on agmatine medium but not on the V8 agar media regardless of light condition or the presence of sucrose, a carbohydrate that induces trichothecene production in/on other media [49].

**Figure 2:**
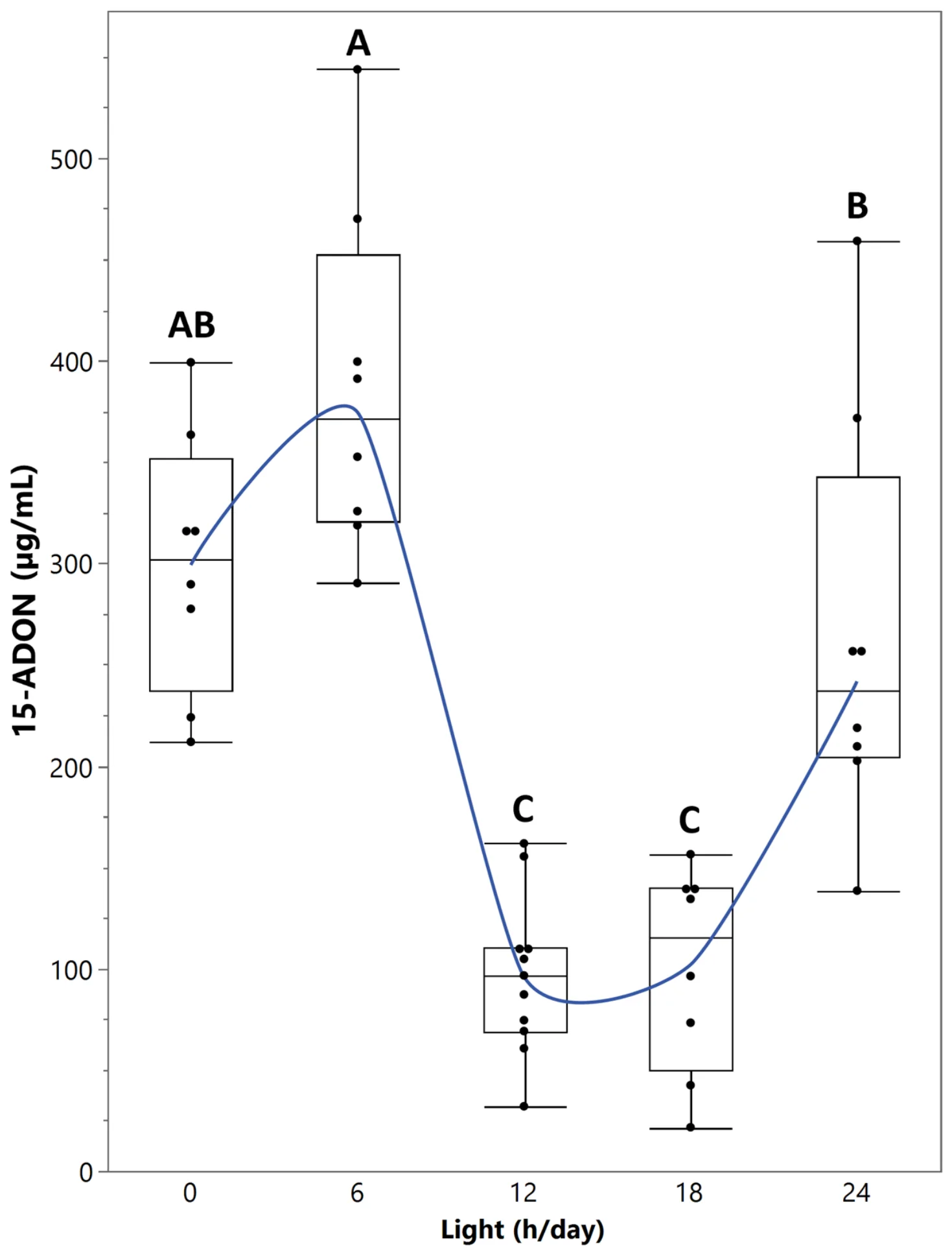
Effect of light duration on 15-ADON production by *Fusarium graminearum* Gz3639 grown on agmatine media. Different letters represent statistically significant differences as determined by a Tukey’s HSD (α = 0.05). The blue line represents the locally weighted kernel regression (calculated by JMP V17.0).

On the agmatine medium, photoperiod significantly altered production of 15-ADON, the only trichothecene detected in this medium (Figure 2). Zero or 6 h light/day caused substantial 15-ADON production, with the highest levels observed under 6 h light/day. Photoperiods of 12 or 18 h light/day suppressed 15-ADON production by 67–75% relative to 0 and 6 h light/day. 15-ADON production was 22% less with continuous light compared with continuous darkness, but the difference was not statistically significant (*p* = 0.8468). Supplementing the fluorescent light spectrum with UV-A radiation had no effect on mycotoxin suppression; *F. graminearum* produced similar levels of 15-ADON under 12 h light/day with or without UV-A (*p* = 0.4008) (Supplemental Figure S1). Since we observed no statistically significant differences in trichothecene production between 0–6 h light/day and 12–18 h light/day, we evaluated only the 0, 12, and 24 h light/day photoperiods for subsequent experiments.

### 2.2. Population, Photoperiod, and Colony Morphology

We evaluated multiple *F. graminearum* strains representing the three most prominent populations of the fungus in North America: NA1, NA2 and NA3. The seven strains selected from each population had the chemotype most often associated with the population: NA1 strains had the 15-ADON chemotype; NA2 strains had the 3-ADON chemotype; and NA3 strains had the NX-2 chemotype.

**Figure 3:**
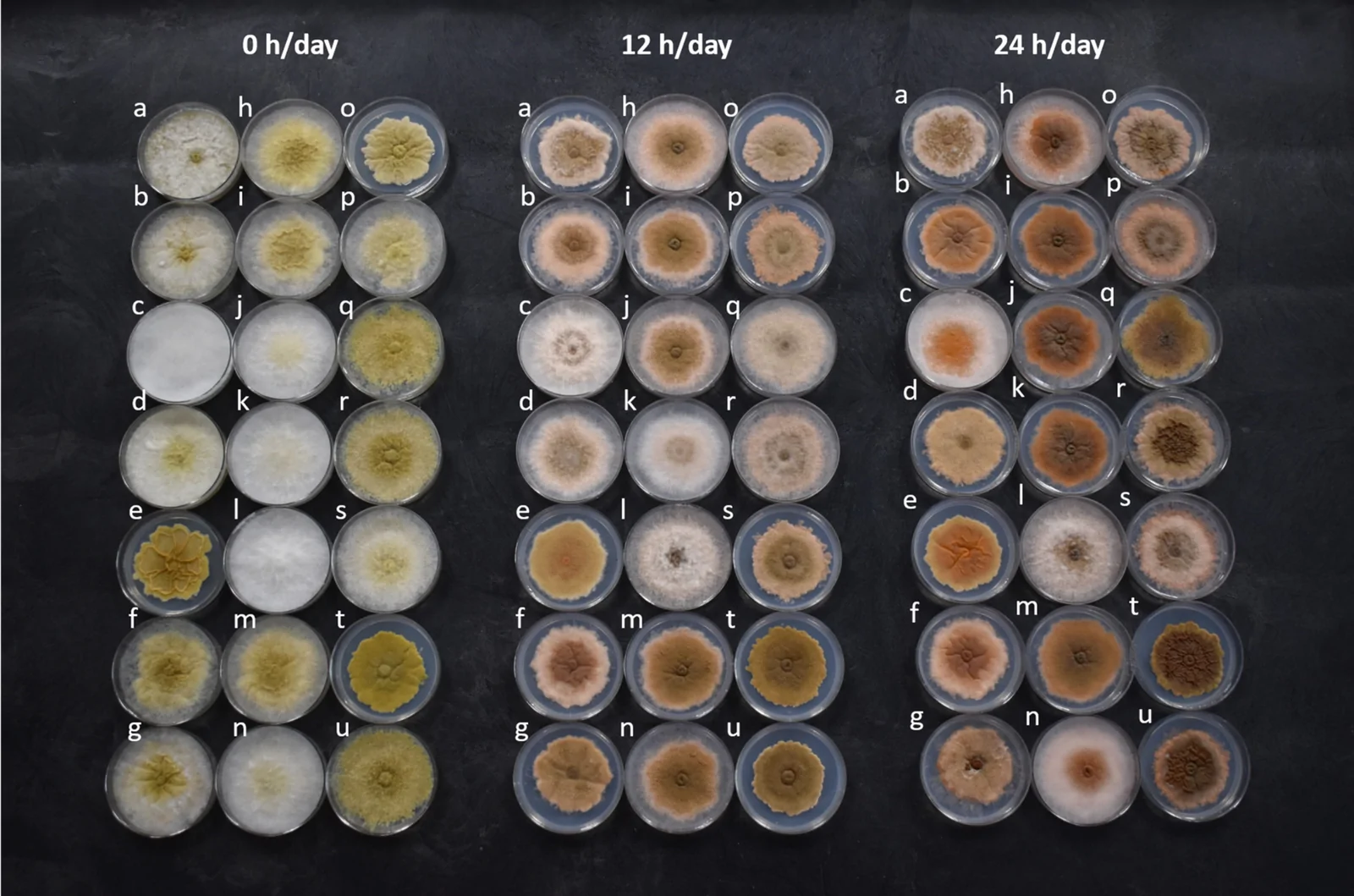
Effect of 0, 12, or 24 h/day of fluorescent light (60 µmol/m^−2^ s) on colony morphology of strains representative of three North American populations (NA1, NA2 and NA3) of *Fusarium graminearum*. NA1 strains: (a) 38762, (b) 38811, (c) 38986, (d) 47571), (e) 52005, (f) 52512, (g) 13MN1-6; NA2 strains: (h) 37525, (i) 38763, (j) 38851, (k) 38964, (l) 46422, (m) 47688, (n) 52008; NA3 strains: (o) 43161, (p) 47659, (q) 43884, (r) 66030, (s) 66031, (t) 66037, (u) 66039. See Supplementary Table S1 for additional strain information.

The *F. graminearum* populations all displayed similar colony morphology and pigmentation in response to variations in photoperiod (Figure 3; Supplemental Figure S2). The *F. graminearum* cultures grown with 0 h light/day developed yellow pigmentation, which may be indicative of carotenoid and aurofusarin accumulation [52]. The *F. graminearum* cultures grown under light 12–24 h/day developed deep red and orange pigmentation, which may be indicative of torulene and rubrofusarin [52]. Cultures grown under dark conditions tended to have more aerial hyphae. Photoperiod affected colony morphology, as mycelia formed fluffier, less dense mats in the dark compared with the denser mycelial mats observed in either 12 or 24 h light/day condition. Additionally, macroconidia were abundant with 12 and 24 h light/day, but were sparse, or absent, in the 0 h light/day condition (Supplemental Figure S3). Ultimately, culture morphology varied widely across strains, with no consistent phenotype due to photoperiod outside of general pigmentation.

### 2.3. Population, Photoperiod and Trichothecene Production

**Figure 4:**
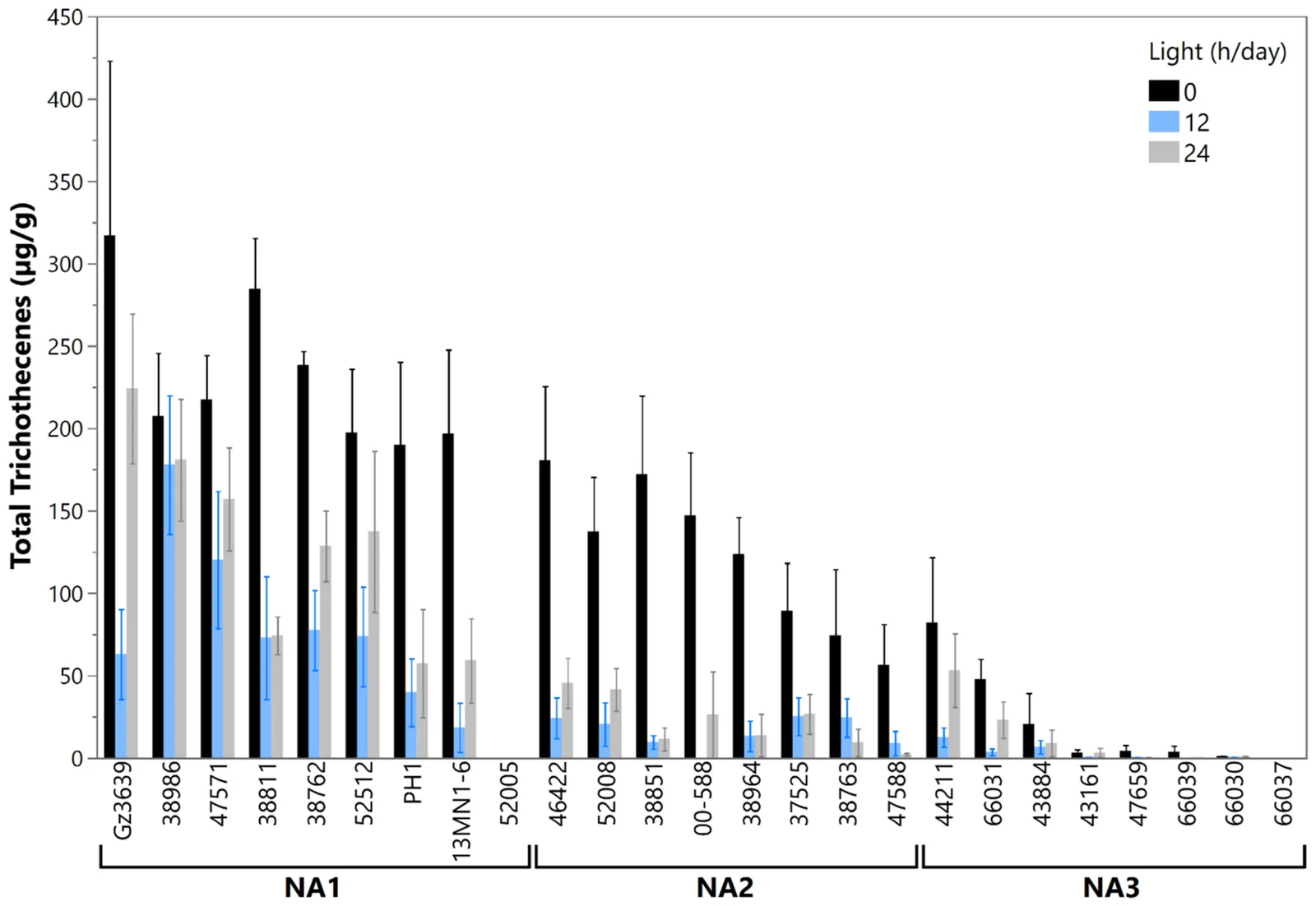
Effect of photoperiod on trichothecene production by strains from three North American populations of *Fusarium graminearum*: NA1, NA2, and NA3. Eight to nine strains from each population were grown on agmatine medium for one week at 25 °C. Photoperiods were 0, 12, and 24 h of fluorescent light (60 µmol m^−2^ s^−1^) per day. Strains 52005 and 66037 did not have detectable trichothecenes (<LOD; signal-to-noise < 3). Error bars represent standard error. See Supplementary Table S1 for additional strain information.

**Figure 5:**
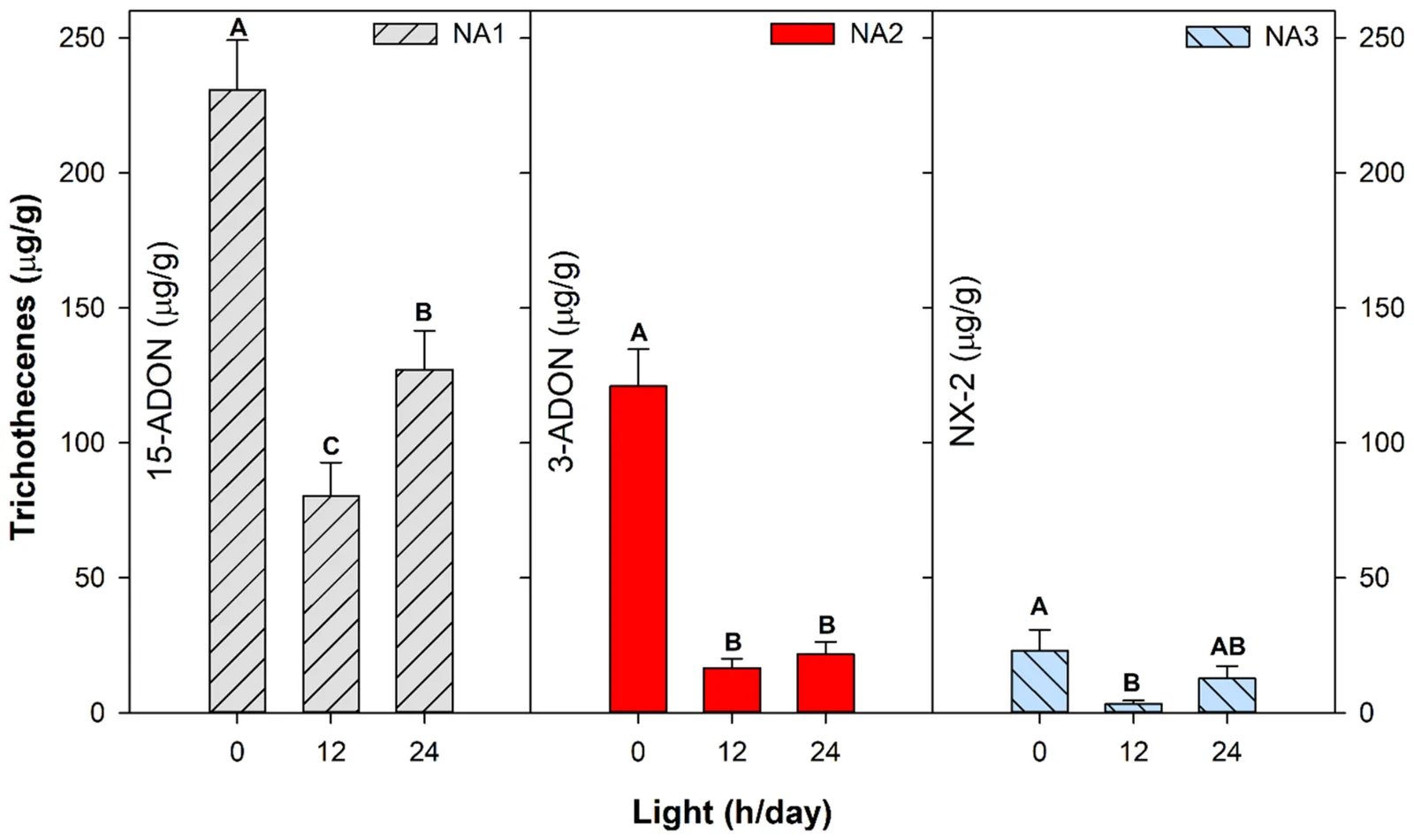
Effect of photoperiod on trichothecene production by strains from three North American populations of *Fusarium graminearum*: NA1, NA2, and NA3. Eight to nine strains from each population were grown on agmatine medium for one week. Values are average levels of trichothecenes for all strains from the same population (Supplementary Table S1). However, data from strains that did not produce detectable levels of trichothecenes under any of the conditions examined (52005 and 66037) were removed from the analysis. NA1 strains included in the analysis produced 15-ADON, NA2 strains produced 3-ADON, and NA3 strains produced NX-2. Within each plot, different letters above the bars indicate statistically significant differences among photoperiod treatments (*n* = 40 for NA1 and NA2; *n* = 35 for NA3) as determined by a non-parametric Wilcoxon test (α = 0.05). Error bars represent standard error.

The effect of photoperiod on trichothecene production was also evaluated in the 25 strains representative of the NA1, NA2, and NA3 populations grown on agmatine medium (Figure 4). Photoperiod had a significant effect on total trichothecene production. Since strains from different populations had different chemotypes, the trichothecene analog(s) detected in cultures of strains from different populations differed. In cultures of NA1 strains, 15-ADON was detected; in cultures of NA2 strains, 3-ADON was detected; and in cultures of NA3 strains, NX-2 was detected. Two strains, 52005 (NA1) and 66037 (NA3), did not produce detectable levels of trichothecenes on agmatine medium with any photoperiod and were removed from subsequent analyses (Figure 5). Excluding the two strains did not alter the statistical interpretation and conclusions of the population comparisons. A post hoc analysis of all trichothecene-producing strains revealed that continuous darkness caused the highest trichothecene production (*p* < 0.0001), while 12 h light/day caused the lowest production (*p* = 0.0169; Supplemental Figure S4). Across all light conditions, strains from different populations produced significantly different levels of trichothecenes on agmatine medium; overall, NA1 strains produced the highest trichothecene levels, NA2 strains produced intermediate levels, and NA3 strains produced the lowest levels (NA1 > NA2 > NA3; *p* < 0.0001). While both NA1 and NA2 strains produced lower levels on agmatine media under 12 and 24 h light/day, photoperiod affected the two populations differently. Relative to dark conditions, trichothecene levels decreased by 64% under 12 h light/day in the NA1 strains and by 85% in the NA2 strains, with NA2 having a significantly greater reduction compared with NA1 (*p* = 0.0466). The NA2 strains also produced fewer trichothecenes under continuous light (83% less) than continuous darkness. The NA1 strains were less affected by photoperiod than the NA2 strains, with only 45% lower trichothecene levels under 24 h light/day (*p* = 0.0009). Unlike the NA1 and NA2 strains, most NA3 strains (43161, 47659, 66039, 66030) had limited trichothecene production on agmatine medium. Again, relative to dark conditions, NA3 strains produced 83% less trichothecene with a 12 h photoperiod but only 51% less under continuous light. Caution should be taken when interpreting these latter results due to the overall low-level induction of trichothecene production in NA3 strains on agmatine medium.

### 2.4. Photoperiod and Trichothecene Production in Seedlings

**Figure 6:**
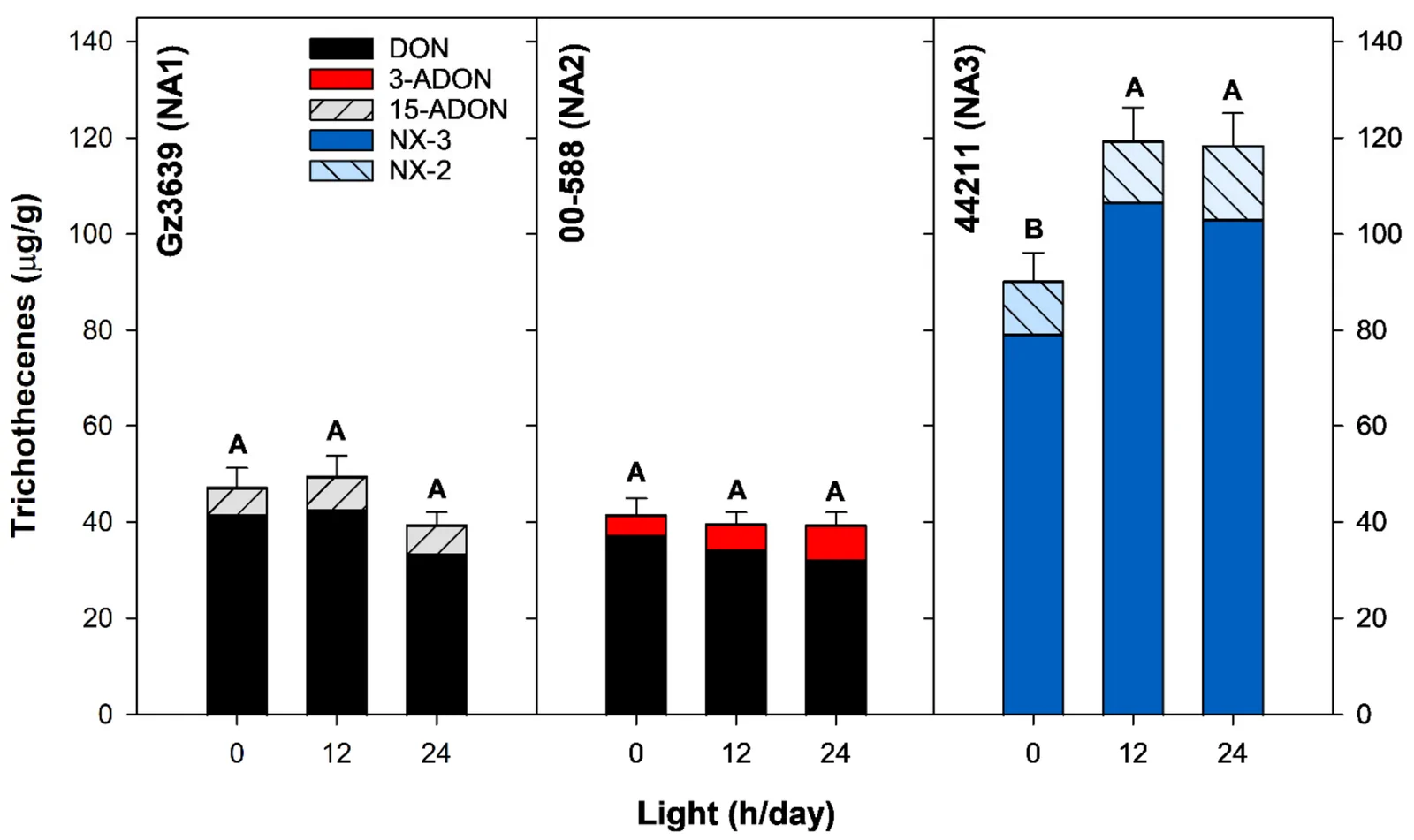
Effect of photoperiod on trichothecene production by strains from three populations of *Fusarium graminearum* grown on wheat seedlings. Photoperiods were 0, 12, and 24 h of fluorescent light (60 µmol m^−2^ s^−1^) per day. One strain from each population was examined: NA1—strain Gz3639, 15-ADON chemotype; NA2—strain 00-588, 3-ADON chemotype; NA3—strain 44211, NX-2 chemotype. Within each plot, different letters above different bars indicate statistically significant differences among photoperiod treatments as determined by Tukey’s HSD test (α = 0.05). Error bars represent standard error.

We also determined whether photoperiod affected trichothecene production *in planta*. The levels and patterns of trichothecene production in inoculated wheat seedlings were markedly different than on agmatine medium (Figure 6). There was a significant strain × photoperiod effect (*p* = 0.0059), where the NA3 strain 44211 produced significantly more trichothecenes than the NA1 (Gz3639) and NA2 (00-588) strains, under all photoperiods. Furthermore, on wheat seedlings, 44211 produced more trichothecenes with 12 and 24 h light/day compared with continuous darkness. This was unlike the agmatine media results, where strains produced the highest levels of trichothecenes with continuous darkness. In wheat seedlings, photoperiod did not affect trichothecene production by the NA1 and NA2 strains. Although agmatine cultures produced only acetylated trichothecenes (15-ADON, 3-ADON, NX-2), in wheat seedlings, the acetylated forms of trichothecenes were mostly deacetylated (~87%), e.g., 15-ADON → DON. There was a significant strain × photoperiod effect on the ratio of acetylated vs. deacetylated trichothecenes (*p* = 0.0014). Under continuous light, the trichothecene analogs produced by the NA1 and NA2 strains had the lowest percent of deacetylation in wheat (*w*/*w*); 84% and 82% of total trichothecenes produced by the NA1 and NA2 strains, respectively, were deacetylated, whereas under continuous darkness, 88% and 90% of all trichothecenes produced by the NA1 and NA2 strains, respectively, were deacetylated. By contrast, photoperiod did not affect percent deacetylation of the trichothecene analog (NX-2) produced by the NA3 strain.

**Figure 7:**
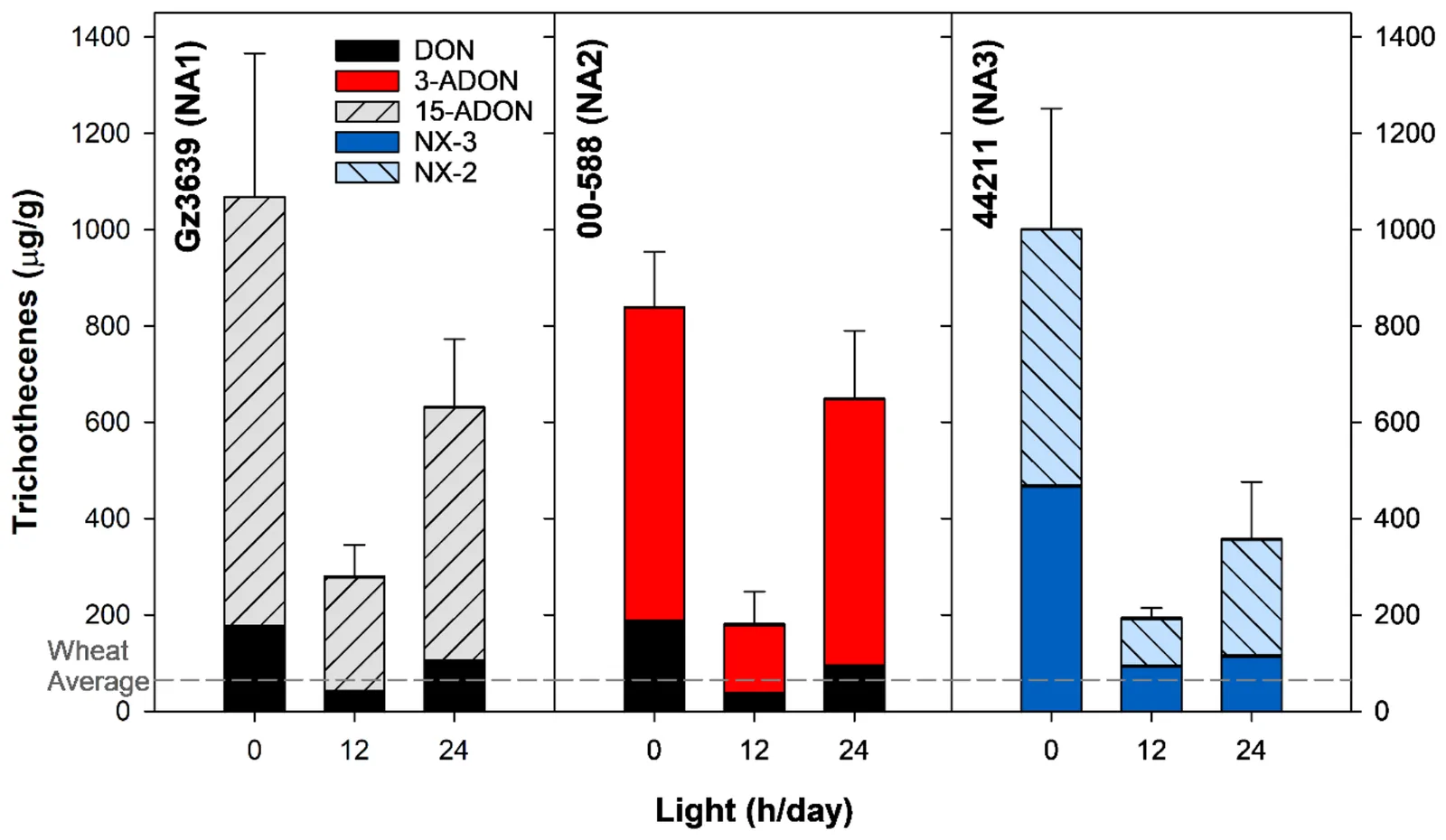
Effect of photoperiod on trichothecene production by strains from three populations of *Fusarium graminearum* grown on corn seedlings. Photoperiods were 0, 12, and 24 h of fluorescent light (60 µmol m^−2^ s^−1^) per day. One strain from each population was examined: NA1—strain Gz3639, 15-ADON chemotype; NA2—strain 00-588, 3-ADON chemotype; NA3—strain 44211, NX-2 chemotype. Error bars represent standard error.

Photoperiod affected trichothecene production by *F. graminearum* in corn seedlings in a manner similar to the effects observed on agmatine medium. That is, in/on both substrates, the highest production occurred under continuous darkness, and markedly lower production occurred under 12 h light/day (Figure 7). The levels of trichothecenes produced by *F. graminearum* in corn seedlings tended to be an order of magnitude higher than those produced on agmatine medium or in wheat seedlings (Figure 5 and Figure 6). The proportion of acetylated versus deacetylated trichothecene analogs also differed in wheat and corn seedlings. In wheat, most of the trichothecenes detected were deacetylated, whereas in corn, most of the trichothecenes detected were acetylated (Figure 7). In inoculated corn seedlings, the proportion of deacetylated trichothecenes also differed by *F. graminearum* strain (*p* < 0.0001) and in response to photoperiod (*p* = 0.0389). In the corn, only 23% of trichothecenes were deacetylated under 24 h light/day, while 32% of trichothecenes were deacetylated under 0 and 12 h light/day. In addition, corn seeds inoculated with strains Gz3639 (NA1) and 00-588 (NA2) had only 18% and 21% deacetylated trichothecene (i.e., DON), respectively. By contrast, corn inoculated with strain 44211 (NA3) had a higher proportion (44%) of the deacetylated trichothecene (i.e., NX-3).

**Figure 8:**
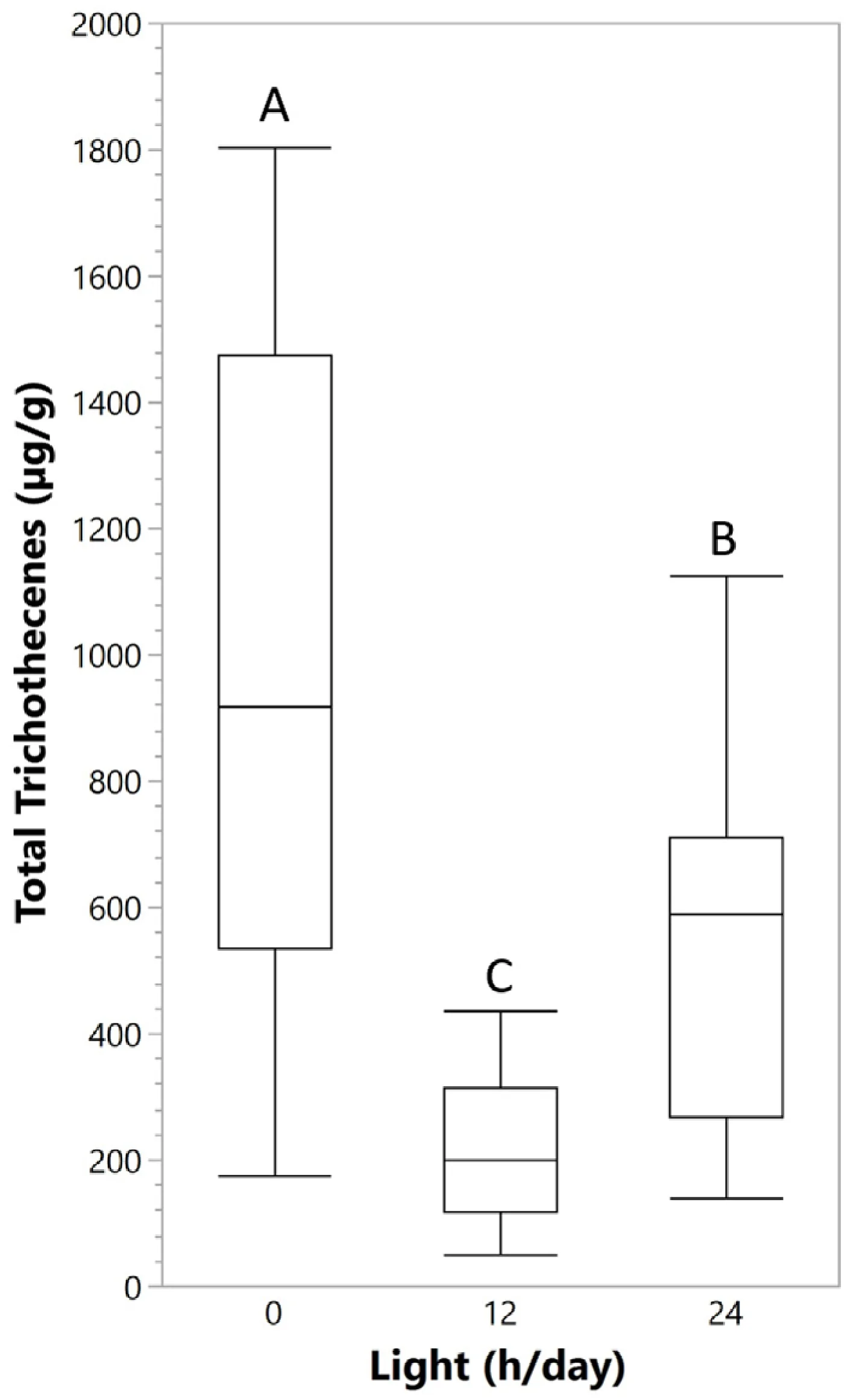
Effect of photoperiod on trichothecene production by *Fusarium graminearum* in corn seedlings. Because no strain effect was observed for total trichothecene production in corn, values shown here are pooled data from the three strains that were evaluated (Gz3639, 00-588 and 44211). In the plot, different letters above the bars indicate statistically significant differences among photoperiod treatments as determined by Tukey’s HSD test (α = 0.05).

Total trichothecene production in the corn seedlings differed substantially by photoperiod (*p* < 0.0001) but not by strain (*p* = 0.5065). Therefore, data were pooled across strains and analyzed with a Tukey’s HSD pairwise test (α = 0.05) to evaluate the impact of photoperiod on *F. graminearum*-infected corn seedlings (Figure 8). Total trichothecene production was greater in corn seedlings than in wheat seedlings or on agmatine medium. Under continuous darkness, 0 h light/day, the sweet corn seedlings had an average trichothecene concentration of 970 µg/g of seed (Figure 8), nearly three orders of magnitude greater than the U.S. FDA advisory level [53]. To determine whether light penetration, and thus photoperiod, would be an important factor in developing corn kernels, light intensity assays were conducted in field-grown corn. On average, corn husk tissue transmitted approximately 8% of incident sunlight (direct sunlight was 1662–1892 µmol m^−2^ s^−1^). By contrast, dark green corn leaf tissue was essentially opaque under the same conditions, with negligible light transmittance (0.4%).

Continuous light also resulted in significant mycotoxin production in corn seedlings, albeit 44% less than in continuous darkness (*p* = 0.0021). The 12 h light/day condition caused the least mycotoxin production in corn, with 87% fewer total trichothecenes than in continuous darkness (Figure 8). Nevertheless, the 12 h light/day treatment resulted in substantially higher levels of trichothecenes in corn seedlings compared with any of the inoculated wheat seedlings.

### 2.5. Photoperiod, Fungal Biomass and Trichothecenes in Cereal Seedlings

**Figure 9:**
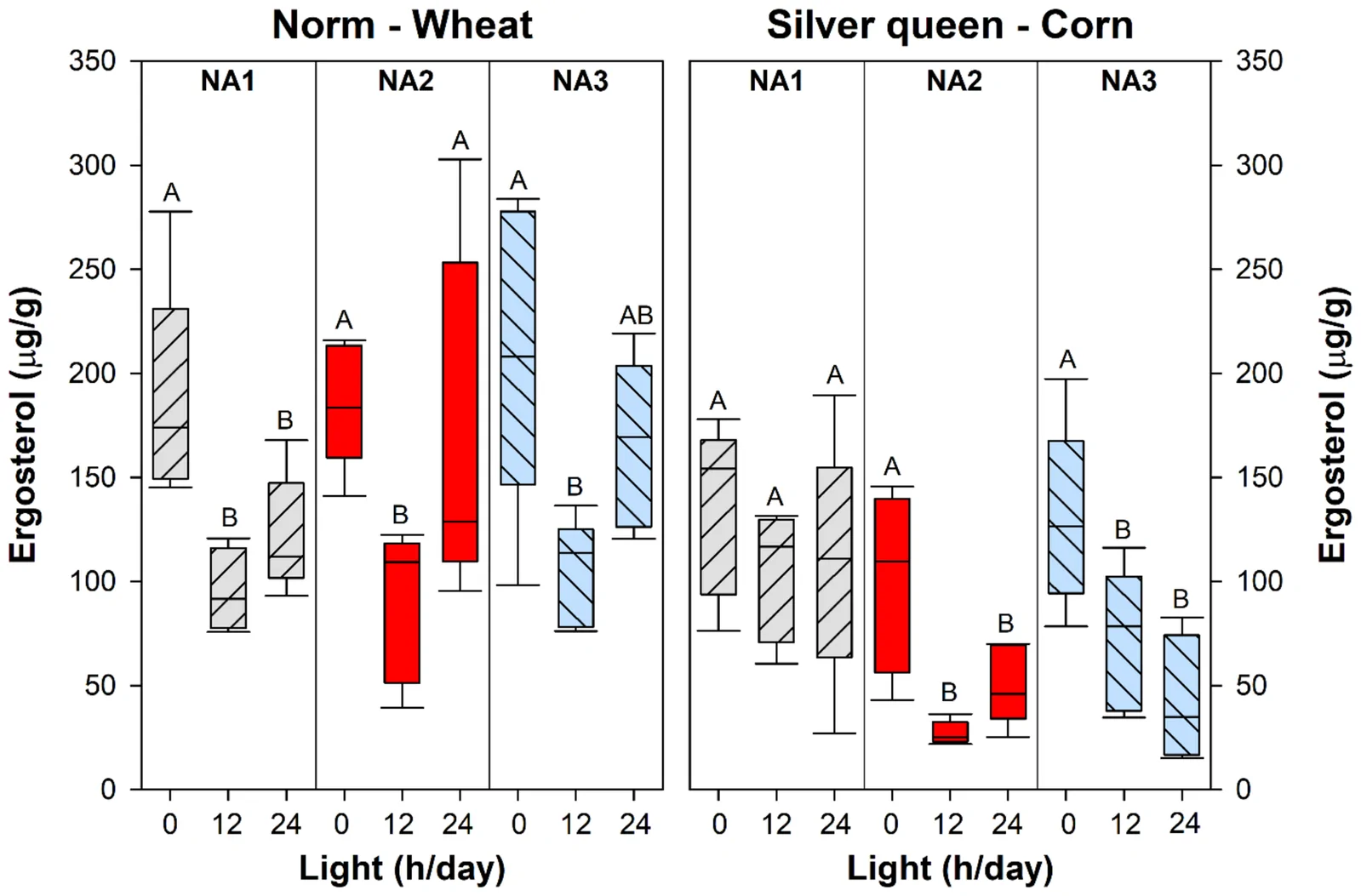
Effect of photoperiod on growth of NA1, NA2 and NA3 strains of *Fusarium graminearum* in wheat and corn seedlings. Light conditions: 0, 12, or 24 h/day of fluorescent light (60 µmol/m^−2^ s). Strains: NA1—Gz3639, NA2—00-588, and NA3—44211. Within individual NA1, NA2 and NA3 plots, different letters above the bars indicate statistically significant differences among photoperiod treatments as determined by Student’s *t* test (α = 0.05).

Growth of *F. graminearum* in corn and wheat seedlings was assessed using estimates of relative biomass as determined by levels of ergosterol, an integral fungal cell membrane lipid, in inoculated seedlings. There was a significant host × strain (*p* = 0.0012) and host × photoperiod (*p* = 0.0485) effect on growth in the inoculated seedlings. On average, growth in wheat seeds was higher than in corn seeds, except for corn seeds inoculated with NA1 (Figure 9). In corn, there were significant differences between strains, with the NA2 strain having the lowest growth. Photoperiod also significantly affected growth in both wheat and sweet corn seedlings. In inoculated wheat seeds, growth was lower under 12 h light/day compared with either 0 or 24 h light/day (Figure 9). In corn, growth was lower in seedlings under 12 h light/day than in dark conditions but was not significantly different than corn seeds under 24 h light/day.

**Figure 10:**
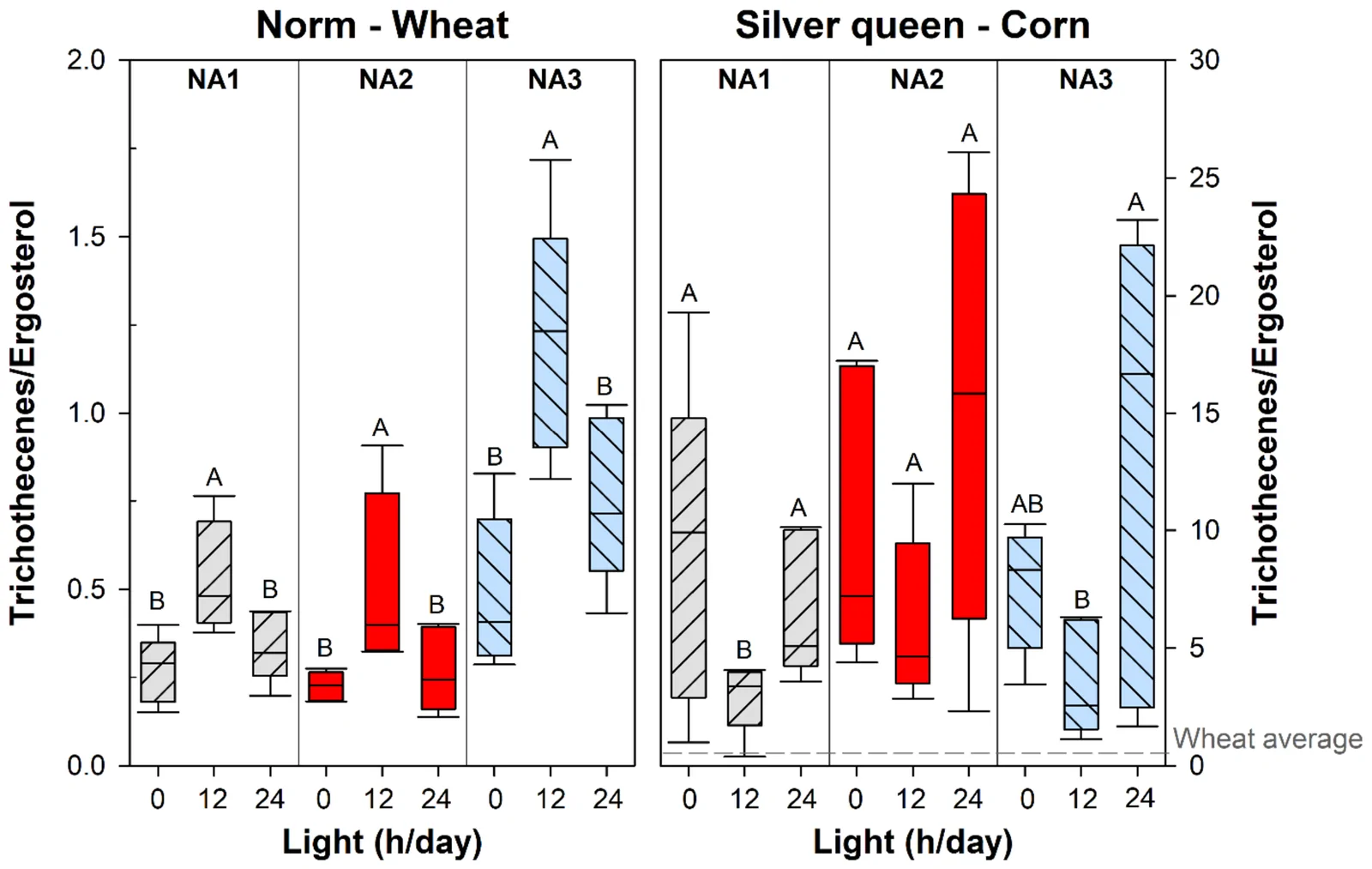
Effect of photoperiod on trichothecene/ergosterol ratios in wheat and corn seedlings inoculated with NA1, NA2 and NA3 strains of *Fusarium graminearum*. Light conditions: 0, 12, or 24 h of fluorescent light (60 µmol m^−2^ s^−1^) per day. *Fusarium graminearum* strains: NA1—Gz3639, NA2—00-588, NA3—44211. Within individual NA1, NA2 and NA3 plots, different letters above the bars indicate statistically significant differences among photoperiod treatments as determined by Student’s *t* test (α = 0.05).

Preliminary comparisons of data from Figure 6, Figure 7 and Figure 9 indicated that trichothecene production was not proportional to growth of *F. graminearum* in corn and wheat seeds. Therefore, we examined the ratio of trichothecene to ergosterol to compare trichothecene production relative to an estimate of fungal growth. There was a significant host × strain (*p* < 0.0001) and host × photoperiod (*p* < 0.0001) effect on the ratio of trichothecene to ergosterol. Trichothecene/ergosterol ratios were 16-fold higher in corn seedlings compared with wheat seedlings (Figure 10). In corn, there were no significant differences in the trichothecene/ergosterol ratio between *F. graminearum* strains. In wheat seedlings, however, the NA3 strain had a significantly higher trichothecene/ergosterol ratio compared with the NA1 and NA2 strains. Photoperiod also significantly affected the trichothecene/ergosterol ratio in wheat and corn seedlings. In the wheat seedlings, 12 h/day of light induced the highest ratio of trichothecenes to ergosterol content across the three *F. graminearum* populations. This effect was not observed in wheat with 24 h light/day, nor was it observed in inoculated corn seedlings. In inoculated corn seedlings, 12 h/day of light suppressed trichothecene production, compared with either 0 or 24 h/day light. These results indicate that the differences in trichothecene production were not solely related to differences in fungal growth.

### 2.6. Photoperiod and Genome-Wide Transcription

**Figure 11:**
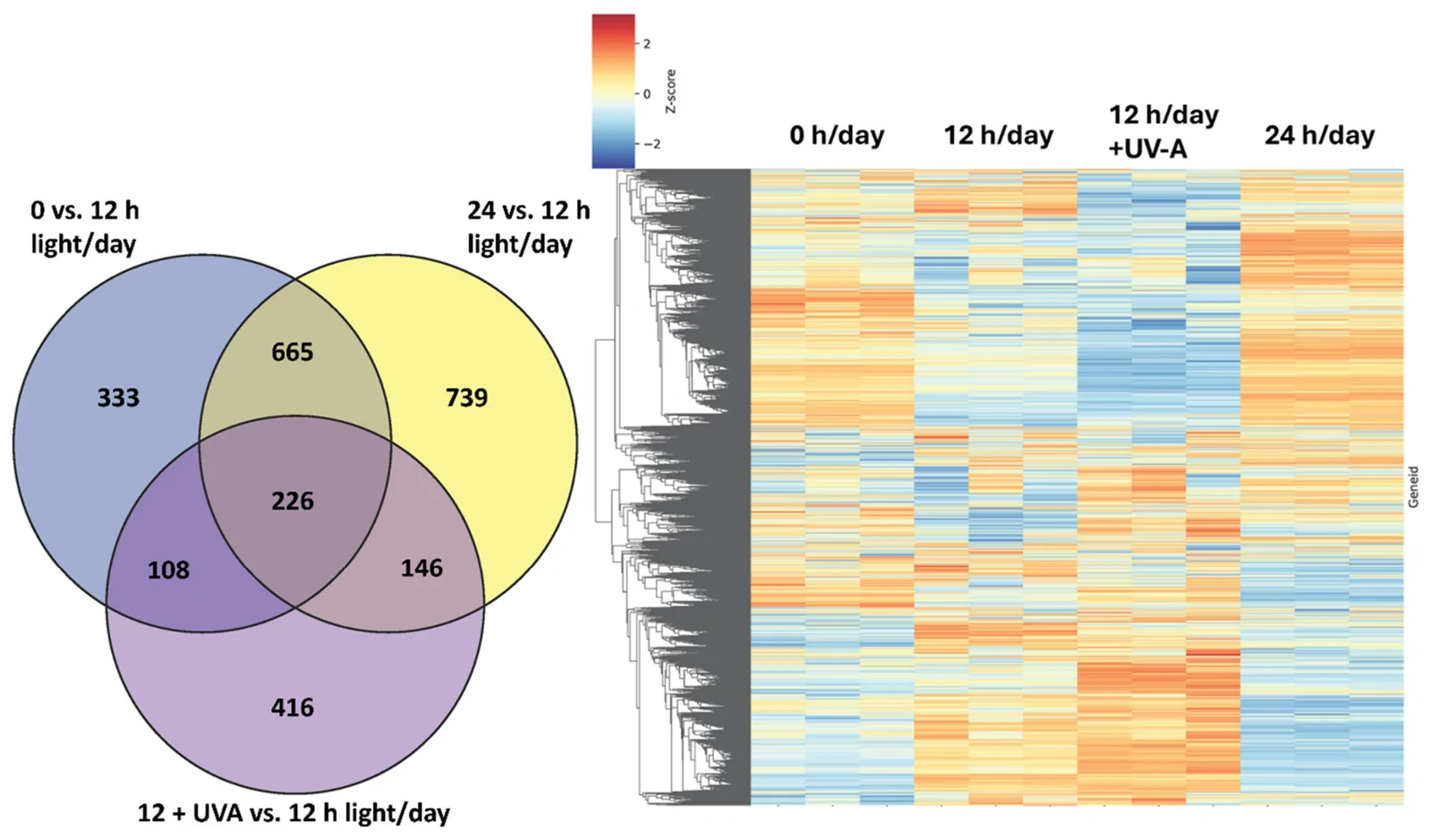
Venn diagram and heatmap showing effects of photoperiod on differential gene expression in the *F. graminearum* strain Gz3639 grown on agmatine medium. Photoperiod treatments: 0 h light/day, 12 h light/day, 12 h light/day + UVA, and 24 h light/day. The Venn diagram illustrates the differentially expressed genes unique to a photoperiod in non-overlapping sections, or shared between photoperiods in overlapping sections, when compared with the gene expression of *F. graminearum* at 12 h light/day. A gene’s z-score is a measure of the number of standard deviations its expression differs from the average expression of the gene across all samples.

We also conducted an RNA-seq analysis to determine how photoperiod affects genome-wide transcription in an NA1 strain of *F. graminearum* (Gz3639) grown on agmatine medium (Figure 11). The 0 and 24 h photoperiods resulted in similar patterns of gene expression and similar numbers of differentially expressed genes (665) compared with the 12 h photoperiod. Although the patterns were similar for the 0 and 24 h photoperiods, there were differences. For example, 333 genes were uniquely differentially expressed in the 0 h photoperiod, but 739 genes were uniquely differentially expressed in the 24 h photoperiod. Supplementation with UV-A also caused significant differences in gene expression, with 416 uniquely differentially expressed genes.

Photoperiod had a significant effect on the expression of some photoreceptor genes. The WCC genes *wcoA* (FGSG_07941) and *wcoB* (FGSG_00710) exhibited modest, but significant, changes in expression, with *wcoA* being slightly downregulated (1.3–1.5-fold) under 12 h light/day compared with 0 and 24 h light/day and *wcoB* being slightly upregulated (1.5-fold) under 0 h light/day compared with the other photoperiods. The blue light flavoprotein receptor gene *lovA* (FGSG_02972) was upregulated (1.4–1.9-fold) under 12 h light/day compared with the other photoperiods, while *lovB* (FGSG_04991) was unaffected by photoperiod. The flavoprotein gene *vvdA* (FGSG_08456) was strongly downregulated (10–12 fold) under 24 h light/day compared with 0 and 12 h light/day and exhibited similar levels of expression under the latter two photoperiods. The *frq* ortholog (FGSG_08069) had negligible expression and no significant response to photoperiod. Expression of the cryptochrome blue light photoreceptor genes *cryD* (FGSG_08852) and *cryP* (FGSG_06765) was inversely correlated with photoperiod length (0 > 12 > 24 h light/day), with significant fold changes ranging from 1.3 to 11. The cryptochrome gene *phrA* (FGSG_00797) was also upregulated (1.7–2.3-fold) in 0 h light/day compared with 12 and 24 h light/day. The rhodopsin green photoreceptor gene *carO* (FGSG_03064) was strongly upregulated under continuous darkness and strongly downregulated under continuous light (16–250-fold). The rhodopsin green photoreceptor gene *opsA* (FGSG_07554) was upregulated under 12 h light/day (1.6–2.3-fold), and the phytochrome red photoreceptor gene *fphA* (FGSG_08608) was upregulated under 0 h light/day (1.6–2.2-fold).

**Figure 12:**
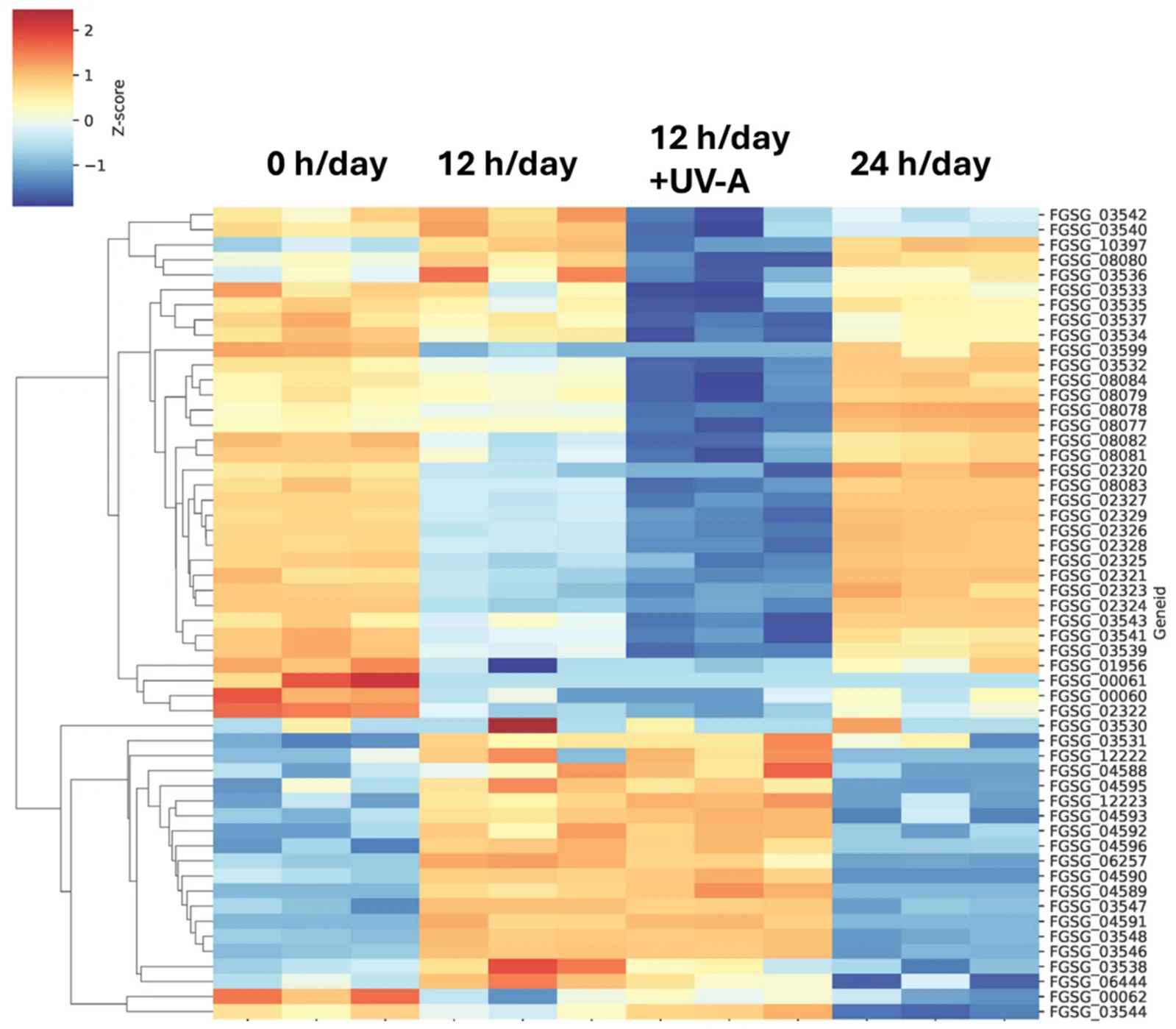
Heatmap showing the effect of photoperiod on the expression of genes associated with pathogenicity and mycotoxin biosynthesis (trichothecenes, aurofusarin, culmorin, butenolide, decalin, Kp4 toxin-like proteins) in the *F. graminearum* strain Gz3639 grown on agmatine medium. Photoperiod treatments: 0 h light/day, 12 h light/day, 12 h light/day + UVA and 24 h light/day. A gene’s z-score is a measure of the number of standard deviations its expression differs from the average expression of the gene across all samples.

Photoperiod also significantly affected the expression of trichothecene biosynthetic genes (*TRI*) in a manner that was consistent with changes in toxin production (Figure 12). When *F. graminearum* was grown under 12 h light/day, as compared with 0 and 24 h light/day, key genes in the trichothecene biosynthesis pathway, *TRI12* (FGSG_03541), *TRI14* (FGSG_03543), and *TRI8* (FGSG_03532), were downregulated (1.3–1.7-fold). The expression of the genes *TRI9* (FGSG_03539) and *TRI13* (FGSG_03542) was upregulated (1.3–1.9-fold) in 12 h light/day, but not with supplemental UV-A. Three genes flanking the *TRI* cluster (FGSG_03546, FGSG_03547, FGSG_03548) were highly induced (17–314-fold) under 12 h light/day relative to the other treatments, including with supplemental UV-A.

The biosynthetic gene cluster associated with the production of decalin-containing diterpenoid pyrones was highly upregulated (3–333-fold) in *F. graminearum* under 12 h light/day; otherwise, transcript levels were nearly absent under the other photoperiods (FGSG_04588; FGSG_04589; FGSG_04590; FGSG_04591; FGSG_04592; FGSG_04593; FGSG_04595; FGSG_04596; FGSG_12222; FGSG_12223). Some aurofusarin biosynthetic genes (FGSG_02324; FGSG_02325) were significantly downregulated (16–18-fold) in 12 h light/day compared with both 0 and 24 h light/day. Similarly, the fusarubin biosynthetic gene (FGSG_09187) was downregulated (2.3–2.6-fold) in 12 h light/day compared with both 0 and 24 h light/day. When *F. graminearum* was grown under dark conditions, Killer Kp4-like genes (FGSG_00060; FGSG_00062), were significantly upregulated (3–10-fold) compared with all other conditions.

**Figure 13:**
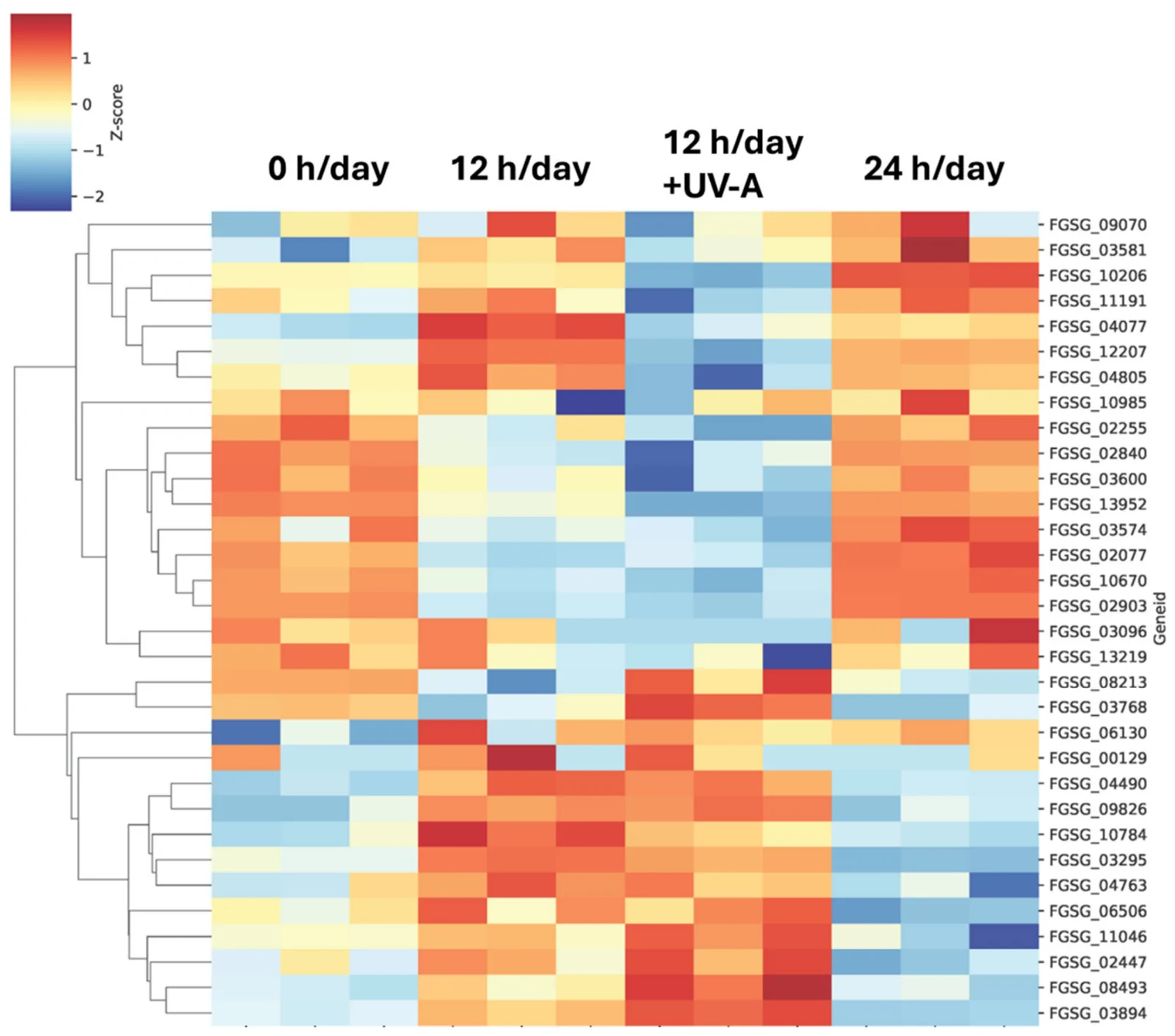
Heatmap showing effects of photoperiod on differential expression of virulence-associated genes in *F. graminearum* grown on agmatine medium. Photoperiod treatments: 0 h light/day, 12 h light/day, 12 h light/day + UVA, and 24 h light/day. A gene’s z-score is a measure of the number of standard deviations its expression differs from the average expression of the gene across all samples.

When evaluating genes identified as virulence effectors, we observed a mosaic of up- and downregulated genes across photoperiod treatments (Figure 13). We employed a network analysis framework to identify genes with high network centrality, or genes that strongly influence the expression of other genes in the photoperiod response and that may have functional importance. Two acetylxylan esterases, FGSG_10670 and FGSG_04077, and one gene encoding a secreted protein (FGSG_03295) were identified as key nodes in the network. FGSG_10670 was significantly downregulated (2.1–2.6-fold), while FGSG_04077 and FGSG_03295 were significantly upregulated (1.5–2.5-fold) in 12 h light/day compared with both 0 and 24 h light/day. Nitric oxide dehydrogenase, (FGSG_04458), was strongly upregulated (8–11-fold) 12 h light/day compared with 0 and 24 h light/day.

## 3. Discussion

The goal of this study was to elucidate the effects of photoperiod on growth and trichothecene production in *F. graminearum* and determine whether the effects vary among three prominent North American populations of the fungus. Our findings indicate that photoperiod affected both growth and trichothecene production, but the effect varied with medium, cereal host, and/or population. Differences in trichothecene levels were not due to differences in the growth of the fungus. Our transcriptomic data analysis of key trichothecene-biosynthesis genes supports the hypothesis that differences in trichothecene levels are due to photoperiod-dependent regulatory factors.

Previous research indicated that light affects mycotoxin production in *F. graminearum* grown on agmatine medium [33]. The *F. graminearum* WCC complex acted as both a light-dependent and independent regulator of trichothecene biosynthesis, causing a light-independent reduction in trichothecene production and strong suppression of biosynthesis with constant light. Our findings were both consistent and inconsistent with the previous literature. We observed a 67–75% decrease in trichothecene levels under 12 and 18 h light/day relative to 0 h/day, but no decrease under 6 h/day. This indicates that longer photoperiods suppress trichothecene production. We posit that the suppression is mediated by circadian photoadaptation, that is, photoreceptor-mediated changes in gene expression resulting from changing light conditions. In our study, the 0 and 24 h light/day photoperiods prevented normal circadian function and allowed us to identify genetic components impacted by circadian regulation by comparing differential gene expression against the 12 h light/day photoperiod. Our transcriptomic analysis of Gz3639 indicated that continuous light suppressed the expression of six photoreceptor genes (*wcoB*, *lovA*, *vvdA*, *phrA*, *opsA*, and *fphA*), which likely inhibited photoadaptation and contributed to a partial recovery of trichothecene production. This effect on trichothecene production was also largely dependent on strain and substrate; we observed differences in trichothecene production among *F. graminearum* populations on agmatine medium and a complete lack of photoperiod response in wheat seedlings but a strong response in corn seedlings. Further, there were photoperiod-induced changes in expression of the WCC genes *wcoA* and *wcoB*, which are homologs of *wc-1* and *wc-2* in *Neurospora crassa* [37,38]. The WCC feedback inhibitor gene encoding *VVDA*, a light-dependent photoreceptor involved with attenuating prolonged light-induced transcription, was strongly downregulated under continuous light, indicating a strong disruption of typical fungal photoreceptor response and transcriptional regulation [54,55].

Fungal circadian cycles are heavily influenced by photoperiod. In *N. crassa*, the core clock components encoded by *wc-1*, *wc-2*, and *frq* drive the central transcription–translation feedback loop that maintains circadian rhythm [36,56]. However, *F. graminearum* lacks the *frq* gene that suppresses WCC activity in the negative feedback loop that controls circadian regulation in many fungi [57]. The expression of the *frq* ortholog (FGSG_08069), previously identified only in the NA1 population [18], was minimal, with no significant effect of photoperiod, indicating that the gene is not involved in light response or circadian regulation in *F. graminearum*. When we evaluated the expression of *F. graminearum* photoreceptor genes, we observed a complex response to photoperiod. In continuous darkness, the cryptochrome (*cryD*, *cryP*, *phrA*), rhodopsin (*carO*), and phytochrome (*fphA*) genes were all significantly upregulated, suggesting these genes are involved in light-independent signaling and/or light-anticipatory pathways that prepare the fungus for daybreak. The strong upregulation of *carO* (FGSG_03064) under continuous darkness was likely due to its co-regulation with the carotenoid pathway, as *carB* (FGSG_03065), *carRA* (FGSG_03066), and *carX* (FGSG_03067) were all similarly upregulated [34].

When *F. graminearum* was grown in a 12 h day/night cycle, *wcoA* and *wcoB* were weakly upregulated, as was the blue light photoreceptor *lovA*, which contributes to photoadaptation during extended light exposure [58]. These gene expression changes suggest a typical circadian response and photoadaptation to changing light conditions. Interestingly, the rhodopsin green light photoreceptor gene *opsA* was also upregulated in 12 h light/day conditions. This gene was previously observed to be moderately upregulated in *F. fujikuroi* with light exposure, and was significantly upregulated during rice infection, but did not influence pathogenicity [41]. Under constant light, most of the photoreceptors or associated genes were downregulated. This is consistent with the strong suppression of photoinduction and is likely the cause of the higher toxin production compared with a 12 h light/day photoperiod.

The effect of photoperiod on trichothecene production differed among *F. graminearum* populations. Photoperiods with 12 h or 24 h light/day suppressed trichothecene production, and this suppression was strongest in NA2 strains. In contrast, only the 12 h light/day photoperiod significantly reduced trichothecene production in NA1 and NA3 strains, with the weakest reduction observed in NA1. Recent findings indicate that in the Canadian provinces of Saskatchewan and Manitoba, there has been a dramatic shift to the 3-ADON chemotype (NA2) [59]. The NA2 population likely has a latitudinal cline, and is more prevalent in northern geographies, which include longer photoperiods in the spring and summer [60]. Our findings suggest that NA2 differs in its response to light compared with the other populations examined, at least with regard to trichothecene production; such population differences warrant further study.

Trichothecene biosynthetic regulators appear to be controlled differently in NA3 compared with NA1 and NA2, as indicated by the distinct differences in trichothecene production levels observed between plant hosts and agmatine medium. Earlier plant-based studies were unable to determine whether differences in trichothecene biosynthesis were due to unique NA3 effectors triggering different host responses or to variation in the regulators that drive trichothecene production in NA3 [18,19]. Our current finding with agmatine supports the hypothesis that trichothecene regulation in NA3 is indeed differentially controlled. In our *in planta* experiments, using a single strain from each of the three populations, the NA3 strain had higher trichothecene production than the NA1 and NA2 strains in wheat seedlings, consistent with previous findings [61]. In corn, NA1, NA2, and NA3 had similar total levels of trichothecene production, but NA3 produced substantially more of the deacetylated form. Additional investigations are needed to fully elucidate the cause of this effect and to identify a suitable alternative to agmatine medium. Testing for NX3 in cereal crops has been limited, but recently, a monoclonal-antibody-based immunoassay was developed for this trichothecene analog [62]. Further characterization of NA3 population prevalence and NX3 contamination levels in U.S. grains would also be prudent.

When evaluating trichothecene production in seedlings, the effect of host species had the greatest impact on trichothecene levels. Total trichothecene production was substantially greater (2–25×) in the corn compared with wheat. This difference was not due to differences in fungal biomass, since average fungal ergosterol concentrations were greater in wheat. Total trichothecene production per unit of ergosterol (trichothecene/ergosterol ratio) was orders of magnitude greater in the corn. This may be due to the differences in host nutritional composition, particularly sucrose content, as sucrose is a known inducer of trichothecene biosynthesis in *F. graminearum*, and the nutritional profile of the host can significantly alter production [48,63]. Photoperiod had a similar effect on trichothecene production in the corn seedlings and on agmatine medium, in that the lowest levels of trichothecene were produced with 12 h light/day, moderate levels were produced with 24 h light/day, and the highest levels were produced with 0 h light/day (=0 h > 24 h > 12 h light/day).

Unlike in corn or on agmatine medium, photoperiod had no effect on the toxin production of tested NA1 and NA2 strains in inoculated wheat seedlings. As a caveat, the current experiment is limited to two standard susceptible wheat and corn cultivars inoculated with a handful of *F. graminearum* strains; broader research is needed to fully elucidate the impact of photoperiod on cereal mycotoxin risk. Our findings are consistent with previous research on barley showing that the presence or absence of light had no effect on DON production in *F. graminearum*-infected barley during the germination step of the malting process [64]. Photoperiod does not appear to be a factor in toxin risk in wheat, at least in the Norm variety, but our findings suggest that the duration of light exposure may influence toxin risk in corn. The reason for this may be quite complex, as it necessarily includes plant response to photoperiod and the production of secondary metabolites, such as the anthocyanins in corn seedlings (Supplementary Figure S5) that may influence *Fusarium* trichothecene production [65,66]. From our light transmission assays, corn husks permit significant incident light penetration, 32–325 µmol m^−2^ s^−1^, that would likely influence *F. graminearum* growth and pathogenicity during ear infection. Total light reaching the corn ear would also depend heavily on canopy structure and environmental factors such as cloud cover. The findings from our study may provide additional factors to incorporate into mycotoxin risk models. Additional research is underway to evaluate the transcriptomic and metabolomic differences in wheat vs. corn and the plant × pathogen response to initial infection.

The acetylation of trichothecenes—especially at the C-3 position—helps prevent *F. graminearum* self-toxicity by reducing toxin affinity to the ribosomal pocket [67,68]. There was a significant host effect on the relative amounts of acetylated/non-acetylated trichothecenes. The deacetylated forms of the trichothecenes (DON, NX-3) found in plants have greater toxicity than their acetylated forms (15-ADON, 3-ADON, NX-2). During plant infection, the percentage of deacetylated trichothecenes was significantly greater in wheat (~82–90%) than in corn (18–32%). Continuous light exposure increased the amount of acetylated DON present in the NA1 and NA2 strains in wheat. The NA3 strain consistently produced a higher amount of deacetylated NX-3 in corn (44%). NX toxins play an essential role in the successful infection and disease spread in wheat, with the phytotoxic NX-3 functioning as a virulence factor with a level of toxicity similar to DON [20,69]. As a caveat, this study was conducted in the seedling stage using representative strains of NA1, NA2, and NA3; to better evaluate this risk, broader studies in mature wheat heads and corn ears using a larger set of *F. graminearum* strains should be conducted.

Differences in trichothecene accumulation were likely the result of photoperiod-dependent gene expression changes to trichothecene biosynthesis genes (e.g., *TRI8*, *TRI9*, *TRI12*, *TRI14*). The *TRI8* gene (FGSG_03532) encodes a deacetylase enzyme that determines whether strains produce either 3-ADON or 15-ADON [70]. The *TRI9* gene (FGSG_03539) encodes a membrane-associated protein associated with increased virulence and trichothecene accumulation [71,72]. The *TRI12* gene (FGSG_03541) encodes a trichothecene efflux pump associated with the secretion of trichothecenes during infection [73]. The *TRI14* (FGSG_03543) gene encodes a cyclization enzyme, which is necessary for the efficient cyclization of trichothecene intermediates isotrichodiol and isotrichotriol [74]. That enzyme, which catalyzes a cyclization reaction early in trichothecene biosynthesis [74], is critical for DON production *in planta* but not in culture and plays a critical role in plant infection [75]. Downregulation of the trichothecene biosynthesis pathway would be expected to reduce toxin accumulation. But expression of *TRI4* (FGSG_03535) and *TRI5* (FGSG_03537), genes essential to the synthesis of trichodiene and the initial oxygenation reactions of trichothecene biosynthesis, did not vary significantly with photoperiod. However, *TRI4* and *TRI5* were downregulated by supplemental UV-A [7,9]. Interestingly, we observed the upregulation of *TRI13* (FGSG_03542) in 12 h light/day but downregulation with supplemental UV-A. *TRI13* is a non-functional pseudogene of the p450 monooxygenase in NA1 strains (e.g., Gz3639) that produce 15-ADON; strains with a functional *TRI13* produce acetylated nivalenol derivatives in culture. That chemotype has been called NIV [76]. None of the *F. graminearum* strains tested in this study produced nivalenol derivatives, but our findings suggest that NIV chemotype strains may produce more trichothecenes under 12 h light/day photoperiods, as *TRI13* is upregulated while most other trichothecene genes are downregulated. To determine whether this is the case, additional studies need to be carried out.

We also observed that three genes flanking the *TRI* gene cluster (FGSG_03546, FGSG_03547, FGSG_03548) were strongly expressed in 12 h light/day (±UV-A). The flanking genes have only predicted functionality, are not involved with trichothecene biosynthesis, and are not significantly upregulated during wheat head infection [77,78]. While it is unclear whether the flanking genes directly influence the suppression of trichothecene accumulation in the 12 h light/day photoperiod, chromatin remodeling in *F. graminearum* has been previously reported to impact the trichothecene gene cluster, altering DON biosynthesis [79]. Regardless, the expression of these *TRI* cluster flanking genes was regulated by photoperiod.

Nearly all trichothecene biosynthetic genes were more strongly downregulated with UV-A light supplementation; however, we did not observe differences in toxin accumulation between 12 h light/day photoperiods with UV-A. Therefore, we can conclude that the suppression of toxin accumulation in these light conditions was due to the circadian effect of the photoperiod rather than the specific wavelength of the UV-A light. It has been previously reported that 385 nm wavelength light can suppress trichothecene biosynthesis and DON accumulation, while a wavelength of 405 nm causes buildup of reactive oxygen species and stimulates DON production [80]. It should be noted that changes in *F. graminearum* trichothecene biosynthesis due to photoperiod occurred on the agmatine medium, which causes an extremely robust induction of genes associated with trichothecene biosynthesis, with 10- to 207-fold greater gene expression than on glutamine media [71]. Substrates that contain polyamines, such as putrescene and agmatine, can strongly induce trichothecene biosynthesis, whereas nitrogen sources such as glutamine do not [50]. As we observed in both agmatine media and corn seedlings, even the 12 h photoperiod had substantial accumulation of trichothecenes, though significantly less than in continuous darkness. Therefore, our observation of moderate downregulation in trichothecene gene expression was consistent with the reduction in toxin production but not its elimination.

We also observed differential expression of genes related to *F. graminearum* toxic secondary metabolites and virulence effectors. The biosynthetic gene cluster for aurofusarin [81], a toxic polyketide-derived naphthoquinone pigment [82], was strongly downregulated in 12 h light/day compared with continuous light or dark. Aurofusarin imparts the yellow-tan pigmentation of *F. graminearum* colonies, especially in dark conditions [52]. Fungi likely produce aurofusarin as a defense against predation, as the pigment is toxic [82], and its production is induced upon mechanical damage to mycelia [83]. The accumulation of aurofusarin was hypothesized to be an antifeedant strategy to protect the fungus. Our results suggest that aurofusarin synthesis was likely suppressed due to a typical photoadaptation response as part of the circadian cycle. Photoperiod-induced changes in aurofusarin, carotenoid, and fusarubin regulation and biosynthesis likely explain the observed differences in colony pigmentation.

The biosynthetic gene cluster that confers production of decalin-containing diterpenoid pyrones was upregulated in 12 h light/day (with or without UVA) but near non-detectable in continuous light or darkness. These secondary metabolites were reported to be virulence factors in the *F. graminearum*–wheat interaction and are upregulated during infection of wheat heads [84]. The changes in expression indicate that this biosynthetic gene cluster is also associated with circadian cycle and fungal response to photoperiod.

While photoperiod impacted expression of numerous genes, including those associated with primary metabolism, we decided to employ a network analysis to identify genes with high network centrality that were associated with toxin biosynthesis or disease virulence. The genes encoding the Killer KP4-like proteins were significantly upregulated in continuous darkness compared with either 12 or 24 h light/day conditions. The *F. graminearum* KP4 proteins are structurally similar to the viral analogs and are associated with increased virulence during root rot infection [85]. But even though the genes encoding KP4 proteins are upregulated during FHB infection, the proteins themselves do not induce cell death in leaves or heads, and knockout mutants did not reduce FHB severity. As the KP4 proteins were associated with root rot infection, a pathosystem characterized by a lack of light, it was consistent that the KP4 genes were upregulated under constant dark conditions.

Among the genes identified as key nodes in the expression network response to photoperiod were two acetylxylan esterase genes, FGSG_10670 and FGSG_04077, and one gene encoding a secreted protein, FGSG_03295. Acetylxylan esterases hydrolyze acetyl groups from xylan, a complex carbohydrate polymer that is a key structural component of plant cell walls [86]. FGSG_03295 is a gene that was previously identified as encoding a secreted protein of unknown function and was preferentially expressed during *F. graminearum* infection of developing maize ears [87]. Although the role these genes play in fungal photoperiod response is unclear, their expression network centrality suggests they are promising targets for future studies.

## 4. Conclusions

This study demonstrated that photoperiod has a profound effect on fungal growth and trichothecene production in *F. graminearum*. This effect varied by substrate (i.e., fungal growth medium or plant host) and among three major North American populations (NA1, NA2, NA3) of the pathogen. Photoperiod had little impact on the toxin production in inoculated wheat seedlings, except for NA3, but a considerable impact on inoculated corn seedlings. Total trichothecene production was substantially greater (2–25×) in inoculated corn compared with wheat; this effect was not due to differences in fungal biomass. Photoperiod also influenced the proportion of deacetylated trichothecenes among the NA1, NA2, and NA3 strains tested. Differences in trichothecene production were likely the result of photoperiod-dependent gene expression changes to trichothecene biosynthesis. However, our current findings are limited to growth medium and cereal seedlings, and additional research is needed to understand how photoperiod affects plant–pathogen interactions in developing wheat heads and corn ears. The results of this study help elucidate the influence of photoperiod on *F. graminearum* and provide insight into how pathogen population differences may influence toxin risk in two major cereal crops.

## 5. Materials and Methods

### 5.1. Fungal Strains

*Fusarium* strains were obtained from the ARS Culture Collection (NRRL) located at the United States Department of Agriculture (USDA), Agricultural Research Service in Peoria, IL. Strains used in preliminary experiments included *F. graminearum* NRRL 22917 (cross reference Gz3639; R. L. Bowden, Kansas State University) [88], *F. graminearum* NRRL 31084 (cross reference CBS 123657, PH-1; Harold Kistler, U.S. Department of Agriculture), *F. graminearum* NRRL 44211 (cross reference S5B-06-4; Randy Clear, Grain Research Laboratory, Canadian Grain Commission, Manitoba, Canada), and *F. graminearum* NRRL 46426 (cross reference 00-588). Further experiments included an additional 21 *F. graminearum* strains selected at random from among the three North American populations (seven strains per population) of the fungus [18,22,60,89,90]. Detailed information about sampled strains can be found in Supplementary Table S1.

### 5.2. Fungal Culturing and Spore Inoculum Production

Strains of *F. graminearum* were removed from storage (−80 °C, 50% glycerol), transferred to water agar (WA; 2% agar), and incubated at 25 °C with 12 h light/day of fluorescent light (60 µmol/m^−2^ s) supplemented with UV-A. After 3 days, one 1 cm^2^ chunk of the resulting agar culture was transferred to V8 agar medium (V8A; 2% agar, 20% V8 juice, 0.3% CaCO_3_), and then incubated at 25 °C with 12 h light/day of fluorescent light (60 µmol/m^−2^ s) supplemented with UV-A. After 3 days, two 1 cm^2^ chunks of the resulting culture were transferred to mung bean broth (MBB; 4% mung bean) and incubated for 3 days in the dark at 28 °C with shaking (200 rpm). The liquid cultures were processed as follows: culture was filtered of solids < 40 µm; culture was centrifuged at 4200 rpm for 10 min; supernatant was decanted, and pellet was resuspended in sterile deionized water (SDI); suspension was centrifuged, and supernatant was decanted; pellet was resuspended in SDI; number of macroconidia in resulting suspension was determined with a hemocytometer; and finally, the suspension was diluted with SDI to a final concentration of 1 × 10^5^ macroconidia/mL. In experiments described below, such suspensions of *F. graminearum* macroconidia are referred to as inoculum.

### 5.3. Light Treatment Assays

Agmatine agar was prepared as reported in [50], amending the medium with 1.14 g/L agmatine sulfate as the sole nitrogen source and 2% *w*/*v* agar. Polystyrene 60 mm × 15 mm petri dishes (VWR; Radnor, PA, USA) were filled with 10 mL of agmatine agar, and then inoculated with 10 µL of spore inoculum pipetted onto the center. Plates were sealed with Parafilm M^®^ (Amcor; Zurich, Switzerland) and incubated under the various conditions for 7 days at 25 °C. Treatment conditions included 0, 6, 12, 18, or 24 h light/day of fluorescent light (60 µmol m^−2^ s^−1^) or a 12 h light/day treatment supplemented with UV-A. Light bulb specifications within growth chambers included F17T8 SPX41, F32T8/TL841, and F17T8-BLB.

Seedling assays utilized *Triticum aestivum*, Norm Spring Wheat (David F. Garvin; Department of Agronomy and Plant Genetics, University of Minnesota) and *Zea mays subspecies saccharata*, Silver Queen Sweet Corn. For each sample, approximately 750 mg of seed was weighed in a 20 mL glass scintillation vial (three corn seeds and fifteen wheat seeds), and then surface sterilized in an aqueous 0.75% sodium hypochlorite solution for 5 min. Equal weight of water to seed was added, and then samples were inoculated with 20 µL inoculum, vortexed to mix, capped loosely with aluminum foil, and stored in their various treatment conditions for 7 days at 25 °C. By day seven, plants had established shoots and rootlets and were, therefore, considered seedlings for our analysis. Treatment conditions included 0, 12, or 24 h/day of fluorescent light (60 µmol/m^−2^ s). Light bulb specifications within growth chambers included F17T8 SPX41 and F32T8/TL841. After incubation, samples were lyophilized for 3 days using a VirTis^®^ Cabinet Lyophilizer MN: 24DX48 GPFD 25L XL-70 (VirTis; Gardiner, NY, USA).

### 5.4. Light Penetration of Corn Husk

Husk tissue was collected from B73 corn plants during pollination. Plants were in experimental field plots at the National Center for Agricultural Utilization Research in Peoria, IL (date collected 7/30/26). The full husk tissue, consisting of 5 layers, was removed from one side of the ear and placed over a quantum meter (Apogee Instruments, Logan, UT, USA; model BQM). Photosynthetic photon flux (µmol m^−2^ s^−1^) was first measured under direct sunlight without plant tissue. A second measurement was taken with the husk tissue covering the sensor. Four biological replicates of husk tissue were evaluated, and the percentage of transmitted light was calculated for each. Leaf tissue was also collected and placed over the quantum meter to determine the percentage of transmitted light.

### 5.5. Trichothecene Extraction and GC-MS Determination

Cultures grown on agmatine medium plates were harvested after 7 days, cut into 1 cm^2^ pieces, transferred to 50 mL polystyrene conical tubes, and extracted with 6 mL of HPLC-grade ethyl acetate with shaking on a vertical vortex for 5 min at 1700 rpm. Afterward, samples were centrifuged at 3000 rpm for 5 min, and the ethyl acetate extracts were dried in 1-dram glass vials under an airstream in a 55 °C heating block. The residues were resuspended in 1 mL ethyl acetate and analyzed with an Agilent 7890 gas chromatograph (GC) fitted with a 30 m HP-5MS column and a 5977 mass detector (MS) (Agilent Technologies; Santa Clara, CA, USA). Samples were introduced with a 20-split injection at 150 °C, the temperature was held for 1 min, then the column was heated at 30 °C/min to 280 °C and held for 1 min. Standard curves of 15-ADON, 3-ADON, and NX-2 were prepared for quantification (10 to 1000 µg/mL; 7-point curve R^2^ > 0.99). Under these conditions, 15-ADON was detected at 7.76 min, 3-ADON at 6.94 min, and NX-2 at 7.00 min.

Lyophilized seedling samples, in their 20 mL glass scintillation vials, were extracted with 4.5 mL of acetonitrile:water (86:14) and shaken at 150 rpm for 24 h on an orbital shaker. A 3 mL aliquot was transferred to a glass 1-dram vial. After drying, 100 µL of a mixture of 1-(trimethylsilyl)imidazole and chlorotrimethylsilane (100:1) was added to the vials. Each vial was quickly vortexed, and then incubated for 30 min. Then, 900 µL of isooctane was added to each vial and briefly vortexed to mix. The reaction was quenched with 1 mL of deionized water and gently mixed. Standard curves of TMS derivatives of DON and NX-3 were prepared (0.3125–80 µg/mL; 9-point curve; R^2^ > 0.99). The organic layer of each sample or standard was analyzed with GC-MS, splitless injection, and the same temperature program as stated previously. Limit of detection and limit of quantification were defined as chromatographic signal-to-noise ratio of 3:1 and chromatographic signal-to-noise ratio of 10:1, respectively.

### 5.6. Cereal Seedlings: Ergosterol Extraction and Quantification

Lyophilized seedlings were extracted using gentle agitation for 2 h in 5 mL ethyl acetate at 25 °C. Extracts were clarified by centrifugation and transferred into 2 mL, 9 mm glass autosampler vials. Ergosterol analyses were performed by high-performance liquid chromatography–tandem mass spectrometry using an Agilent 1100 LC (Agilent Technologies; Santa Clara, CA, USA) coupled to a SCIEX 3500 triple quadrupole instrument (SCIEX; Vaughan, ON, Canada) fitted with an atmospheric pressure chemical ionization (APCI) source operating in positive ionization mode. Separations were accomplished in a 0.4 mL/min effluent flow using a 25 min 80–100% gradient of methanol against water (both modified with 0.3% acetic acid) on a Kinetex C18 column (50 mm length × 2.1 mm diameter, 2.6 µm particle size, Phenomenex, Torrance, CA, USA) heated in a column oven at 40 °C. The APCI source was operated using an ionization temperature of 450 °C. MS/MS detection was done in multiple reaction monitoring (MRM) mode using the following instrument parameters: ionization gas flow 1 (GS1): 28; ionization gas flow 2 (GS2): 60; collision gas flow (CAD): 7; ion corona current (NC): 3; de-clustering potential (DP): 81 V; entrance potential (EP): 81. Two MRM transitions were used for monitoring of the [M+H]^+^ ion of ergosterol. The “quantifier” MRM transition monitored ions going from 379.3 *m*/*z* to 69.2 *m*/*z* utilizing a collision energy (CE) of 49 V and a collision cell exit potential (CXP) of 10 V. Similarly, the “qualifier” MRM transition monitored ions going from 379.3 *m*/*z* to 159.1 *m*/*z* utilizing a CE of 29 V and a CXP of 4 V. SCIEX Analyst software (version 1.7.2; Build # 8287) was used to control the coupled chromatography–mass spectrometry instrument and to process the collected data files for LC-MS/MS experiments. Quantifications were based on integrated intensities from the quantifier MRM transition upon confirmation with observation of a corresponding qualifier MRM transition. Standard curves for ergosterol were prepared (1.2–120 µg/mL; 11-point curve; R^2^ > 0.98), and the interday %RSD was less than 10 for the highest-concentration sample (120 µg/mL). Limit of detection and limit of quantification were defined as chromatographic signal-to-noise ratio of 3:1 and chromatographic signal-to-noise ratio of 10:1, respectively. HPLC-grade methanol, acetonitrile, ethyl acetate and acetic acid were purchased from Thermo Fisher Scientific (Carlsbad, CA, USA). The ergosterol standard was purchased from Sigma-Aldrich (Carlsbad, CA, USA).

### 5.7. Scanning Electron Microscopy

At 4 days of growth on agmatine media, 1 cm^2^ chunk agar was cut from the leading edge of *F. graminearum* 44211 grown under the varied light treatment conditions. Tissue pieces were first submerged in 8% glutaraldehyde for 6 h, and then fixed by exposure to osmium tetroxide vapor over a 24 h period. Fixed specimens were dehydrated with an ethanol series (1 h per step: sterile deionized water, 25% ethanol, 50% ethanol, 75% ethanol, 95% ethanol, 100% ethanol) and stored in 100% ethanol until use. Dehydrated specimens were critical-point dried using a Samri-PVT Critical Point Dryer (Tousimis; Rockville, MD, USA) and mounted onto 12 mm SEM stubs using double-sided carbon tape (SPI Supplies, Structure Probe Inc.; West Chester, PA, USA). Mounted specimens were sputter coated in a gold and palladium mixture using a SPI-Module Sputter/Carbon Coater (SPI Supplies, Structure Probe Inc.; West Chester, PA, USA) and viewed using a JOEL JSM-6010LA scanning electron microscope (JEOL USA Inc.; Peabody, MA, USA).

### 5.8. RNA Extraction and Library Preparation

After 7 days of growth on agmatine medium, Gz3639 fungal cultures were flash frozen using liquid nitrogen. Treatment conditions included 0, 12, 12 + UV-A, or 24 h/day of fluorescent light (60 µmol/m^−2^ s). Cultures grown under 12 h light/day (±UV-A) were collected 6 h into the light cycle. Total RNA was isolated from approximately 40–55 mg lyophilized, ground tissue using a combination of the Trizol reagent (Life Technologies, Carlsbad, CA, USA) and the RNeasy Mini Kit (Qiagen, Germantown, MD, USA) extraction methods following standard manufacturer protocols with an additional DNase step using the RNase Free DNase Kit (Qiagen, Germantown, MD, USA). RNA quantity and quality were initially assessed by evaluating the 260/280 and 260/230 values using a Nanodrop 8000 Spectrophotometer (Thermo Fisher Scientific; Waltham, MA, USA). Sample RNA Integrity Number (RIN) was determined using an Agilent Technologies 2200 Tape Station (Agilent Technologies; Santa Clara, CA, USA). The RIN values of 8–10 were considered intact RNA, 6–7 acceptable, 4–5 partially degraded, and <4 highly degraded. Quality thresholds for all samples in this experiment were 260/280 > 2.00, 260/230 > 1.70, and RIN values ≥ 8.0. Sample RIN values averaged 8.4, with a range of 8.0–9.4.

Final RNA quantification was done using a Qubit 4 Fluorometer (Invitrogen, Thermo Fisher Scientific; Carlsbad, CA, USA) to ensure accurate dilution calculations for library preparation. cDNA libraries were then created using Illumina’s Stranded mRNA Prep kit (20040532) per manufacturer’s instructions. Libraries were sequenced on a NextSeq 2000 (Illumina Inc., San Diego, CA, USA), using a P1 flow cell kit run at 2 × 100 cycles paired end.

### 5.9. Transcriptomic Analysis

Splice site and exon coordinates were extracted from the reference genome sequence of *F. graminearum* (strain PH-1, NCBI accession GCF_000240135.3) using HISAT2 v2.2.1 utility scripts [91]. HISAT2 was also used to index the reference genome and align RNA-seq reads. SAM files were converted to BAM format, sorted, and indexed using SAMtools v1.17 [92]. A gene-level count matrix was generated with featureCounts in Subread v2.0.8 [93,94]. Differential expression analysis was conducted using a negative binomial generalized linear model (GLM) with Cook’s distance outlier detection implemented in PyDESeq2 v0.5.0 [95]. Statistical significance was assessed with Wald tests and Benjamini–Hochberg false discovery rate correction. Heat maps were generated from log1p-transformed normalized counts with gene-wise z-score scaling applied across samples for selected genes of interest and visualized using seaborn v0.13.2 in Python v3.12.8. Venn diagrams of shared and unique differentially expressed genes were generated with VennDiagram v1.7.3 in R v4.3.2.

### 5.10. Statistical Analysis

Data were first evaluated for normality using a Shapiro–Wilk test (JMP V17.0). If normality was found (*p* > 0.05), then a Tukey’s HSD test (α = 0.05) was performed to evaluate differences in trichothecene accumulation. Within-strain differences, the effect of UV-A radiation, and differences in mycotoxin production ratio were evaluated using a Student’s *t* test (α = 0.05). Percentages were arc sin transformed before analysis. When a normality assumption was not met, data were either square root transformed, or a non-parametric Wilcoxon/Kruskal–Wallis test (α = 0.05) was used to determine significant differences in mycotoxin accumulation.

## Data Availability

The original contributions presented in this study are included in the article/Supplementary Material. Further inquiries can be directed to the corresponding author.

## Supplementary Materials

The following supporting information can be downloaded at: https://www.mdpi.com/article/10.3390/toxins18100459/s1, Table S1: List of *Fusarium* strains used in the manuscript; Figure S1: Effect of photoperiod and UV-A supplementation on 15-ADON production by *Fusarium graminearum* Gz3639 grown on agmatine media; Figure S2: Effect of photoperiod on *Fusarium* physiology; Figure S3: Scanning electron micrographs of *Fusarium* morphology; Figure S4: Effect of photoperiod on trichothecene production from *Fusarium graminearum* populations (NA1, NA2, NA3) grown on agmatine media; Figure S5: Wheat (Norm) and corn (Silver Queen) controls; Figure S6: Representative samples of *Fusarium*-inoculated corn and wheat after one week at 0, 12, or 24 h/day of fluorescent light (60 µmol/m^−2^ s) at 25 °C.
