# Supplementary material for: Effect of Photoperiod on Growth and Production of Trichothecene Mycotoxins in Fusarium graminearum
Source: Toxins (Basel). 2026 Oct 21;18(10):459. doi: 10.3390/toxins18100459 (PMC13709573; doi:10.3390/toxins18100459)
Supplement: Supplementary file 1 [file toxins-18-00459-s001.zip › toxins-4605253-supplementary.pdf]

Supplementary Materials: Effect of Photoperiod on Growth and Production of Trichothecene Mycotoxins in *Fusarium graminearum*

William T. Hay, Susan P. McCormick, Nathan D. Kemp, Mark Busman, Daren W. Brown, Hye-Seon Kim, Carson M. Andorf, Stephen F. Harding, Robert H. Proctor and Martha M. Vaughan

Table S1. List of *Fusarium* strains used in the manuscript. *F. graminearum* strains were chosen from among those tested in Laraba et al. 2023 [19]. a) Strains were received and are available from the U.S. Department of Agriculture, Agriculture Research Service NRRL Culture Collection in Peoria, Illinois. b) Strains were assigned to the genetic populations NA1, NA2, and NA3 using SNP genotyping [18]. c) Strain chemotypes were determined via multilocus genotyping or gas chromatography-mass spectrometry screening [18,22,60,89,90].

Fusarium species	NRRL Identifier (a)	Equivalent Identifier	Population (b)	Chemotype (c)	Geographic Location	Crop Host	Reference
graminearum	22917	Gz3639	NA1	15ADON	Kansas, U.S.A.	Wheat	Proctor et al. 1995 [88]
graminearum	31084	PH1	NA1	15ADON	Michigan, U.S.A.	Corn	Kelly and Ward 2018[18]
graminearum	38762	Q-05-16	NA1	15ADON	Quebec, Canada	Wheat	Kelly et al. 2015[90]
graminearum	38811	Q-05-65	NA1	15ADON	Quebec, Canada	Wheat	Kelly et al. 2015[90]
graminearum	38986	ON-05-114	NA1	15ADON	Ontario, Canada	Wheat	Kelly et al. 2015[90]
graminearum	47571	Q-07-4	NA1	15ADON	Quebec, Canada	Wheat	Kelly et al. 2015[90]
graminearum	52005	M6-07-28	NA1	15ADON	Manitoba, Canada	Wheat	Kelly et al. 2015[90]
graminearum	52512	A2-07-91	NA1	15ADON	Alberta, Canada	Wheat	Kelly et al. 2015[90]
graminearum	64387	13MN1-6	NA1	15ADON	Minnesota, U.S.A.	Wheat	Liang et al. 2015[60]
graminearum	37525	S8A-04-2	NA2	3ADON	Saskatchewan, Canada	Wheat	Ward et al. 2008[18]
graminearum	38763	Q-05-17	NA2	3ADON	Quebec, Canada	Wheat	Kelly et al. 2015[90]

graminearum	38851	Q-05-105	NA2	3ADON	Quebec, Canada	Wheat	Kelly et al. 2015[90]
graminearum	38964	ON-05-92	NA2	3ADON	Ontario, Canada	Wheat	Kelly et al. 2015[90]
graminearum	38980	ON-05-108	NA2	3ADON	Ontario, Canada	Wheat	Kelly et al. 2015[90]
graminearum	46422	00-566	NA2	3ADON	Ontario, Canada	Wheat	Kelly et al. 2015[90]
graminearum	46426	00-588	NA2	3ADON	Minnesota, U.S.A.	Wheat	Gale et al. 2007
graminearum	47588	Q-07-21	NA2	3ADON	Quebec, Canada	Wheat	Kelly et al. 2015[90]
graminearum	43161	M11-05-oat4	NA3	NX-2	Manitoba, Canada	Oat	Kelly et al. 2015[90]
graminearum	43884	ON-06-4	NA3	NX-2	Ontario, Canada	Wheat	Kelly et al. 2015[90]
graminearum	44211	S5B-06-4	NA3	NX-2	Saskatchewan, Canada	Wheat	Kelly et al. 2015[90]
graminearum	47659	Q-07-92	NA3	NX-2	Quebec, Canada	Wheat	Kelly et al. 2015[90]
graminearum	66030	06-146	NA3	NX-2	Minnesota, U.S.A.	Wheat	Liang et al. 2015[60]
graminearum	66031	12MN1-3	NA3	NX-2	Minnesota, U.S.A.	Wheat	Liang et al. 2015[60]
graminearum	66037	02-264	NA3	NX-2	Minnesota, U.S.A.	Wheat	Liang et al. 2015[60]
graminearum	66039	02-267	NA3	NX-2	Minnesota, U.S.A.	Wheat	Liang et al. 2015[60]
verticillioides	20956	M-3125		Fumonisin	California U.S.A.	Corn	

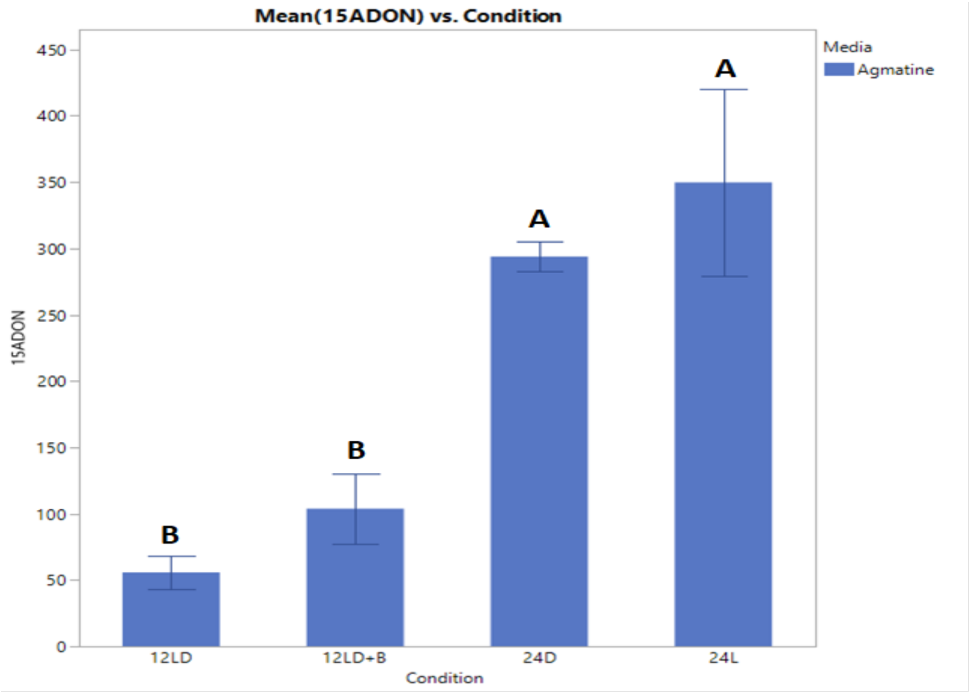

Figure S1. Effect of photoperiod and UV-A supplementation on 15ADON production by *Fusarium graminearum* Gz3639 grown on agmatine media. Different letters represent statistically significant differences as determined by a Student's t test (α = 0.05).

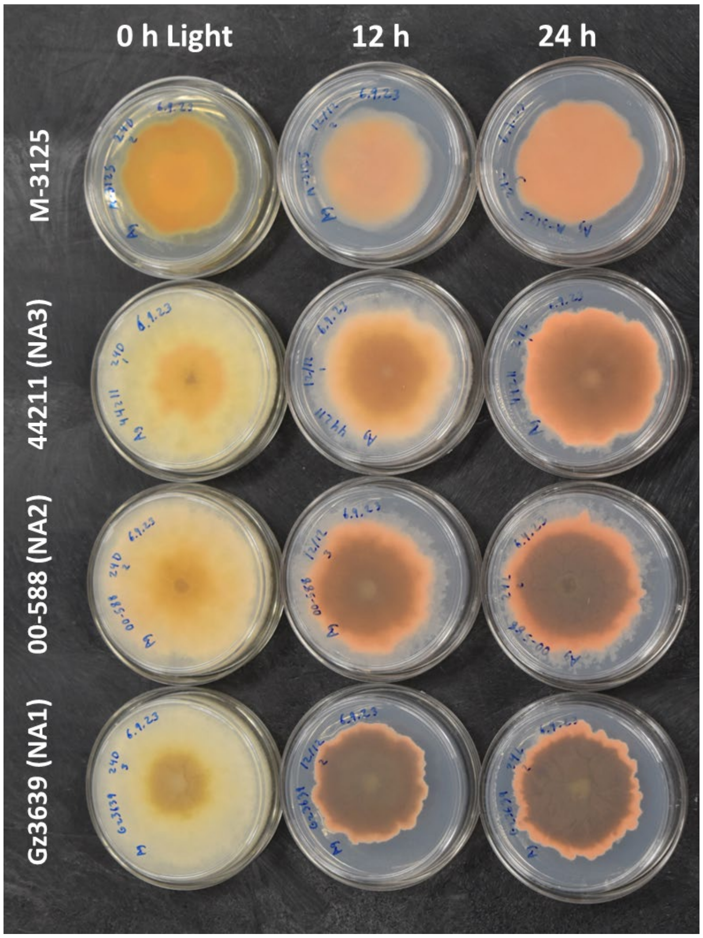

Figure S2. Effect of photoperiod on *Fusarium* physiology – bottom of the plate showing pigment deposition. *Fusarium graminearum* populations: Gz3639 (NA1), 00-588 (NA2), 44211 (NA3), and *Fusarium verticillioides* M-3125.

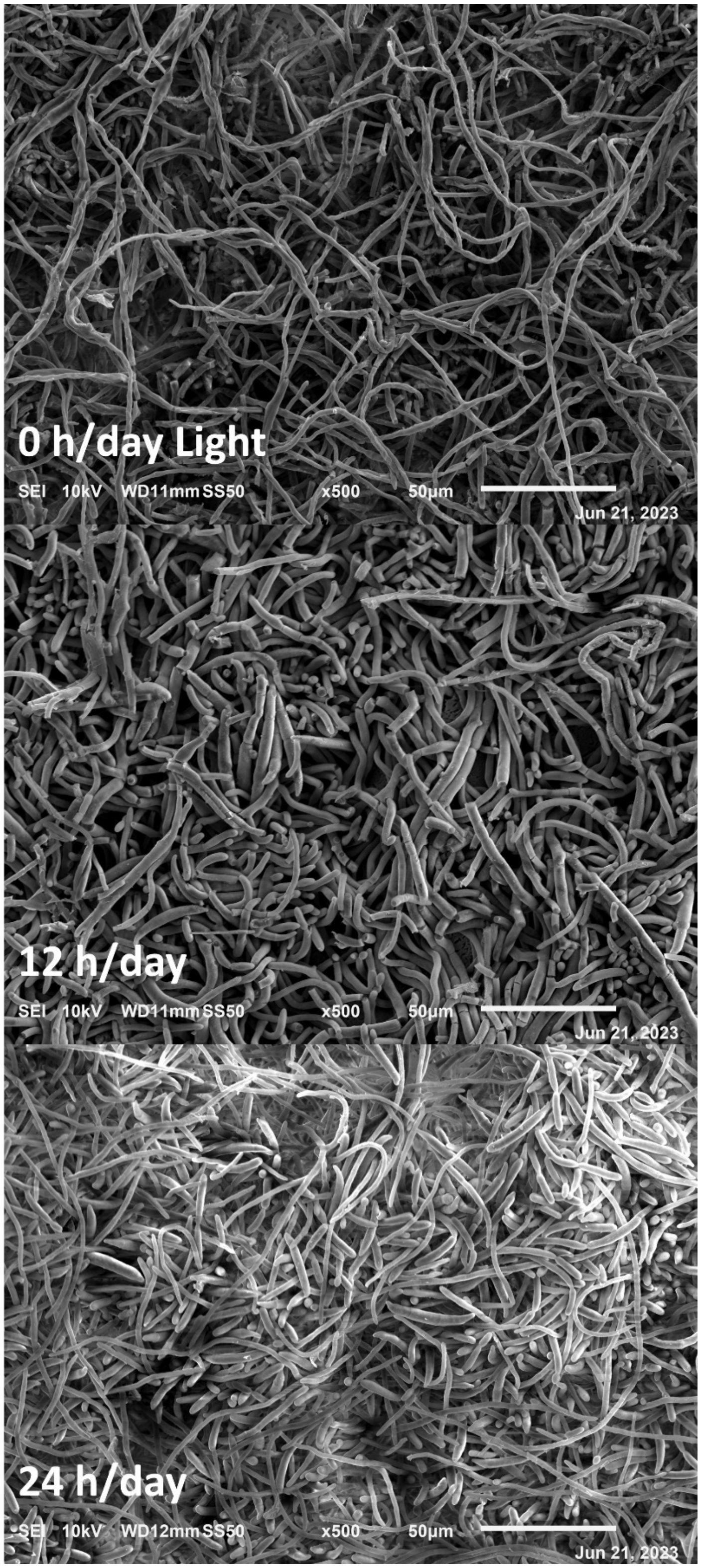
0 h/day Light
SEI 10kV WD11mm SS50 x500 50μm
Jun 21, 2023
12 h/day
SEI 10kV WD11mm SS50 x500 50μm
Jun 21, 2023
24 h/day
SEI 10kV WD12mm SS50 x500 50μm
Jun 21, 2023

Figure S3. Scanning electron micrographs of *Fusarium* morphology. Fungus grown on agmatine media with 0, 12, or 24 h/day of fluorescent light (60 µmol/m^{-2} sec) at 25 °C. Images taken 3 days after inoculation.

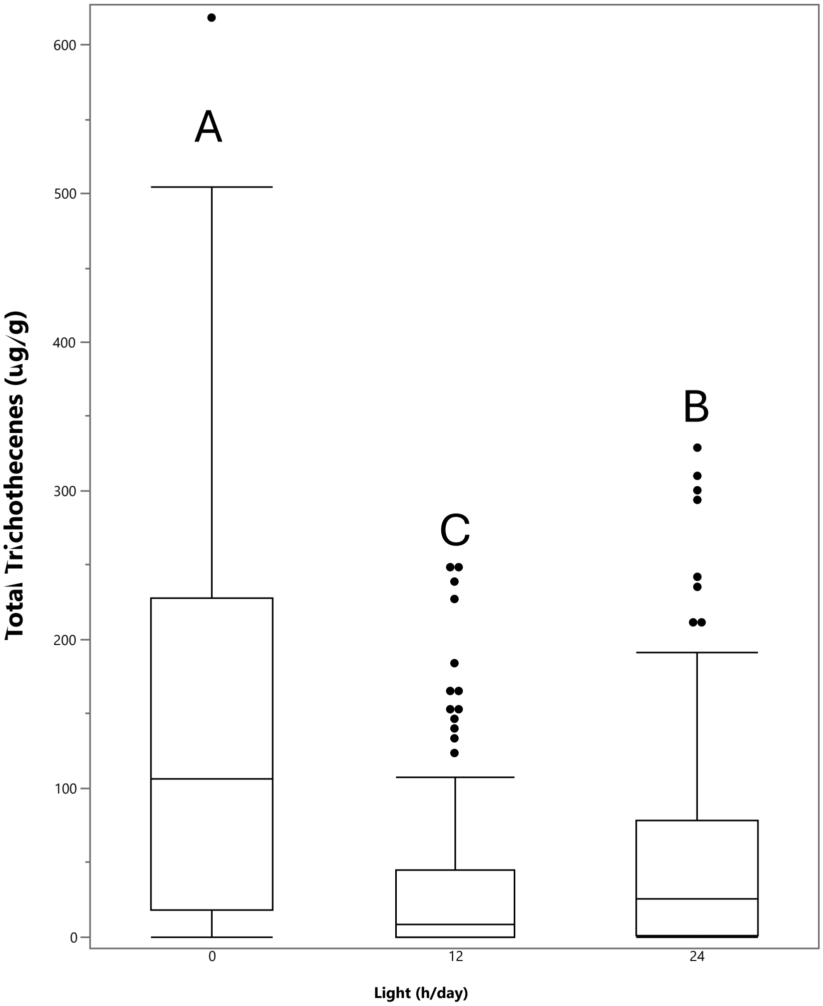

Figure S4. Effect of photoperiod on trichothecene production from *Fusarium graminearum* populations (NA1, NA2, NA3) grown on agmatine media. Letters represent statistically significant differences as determined by a non-parametric Wilcoxon test (α =0.05).

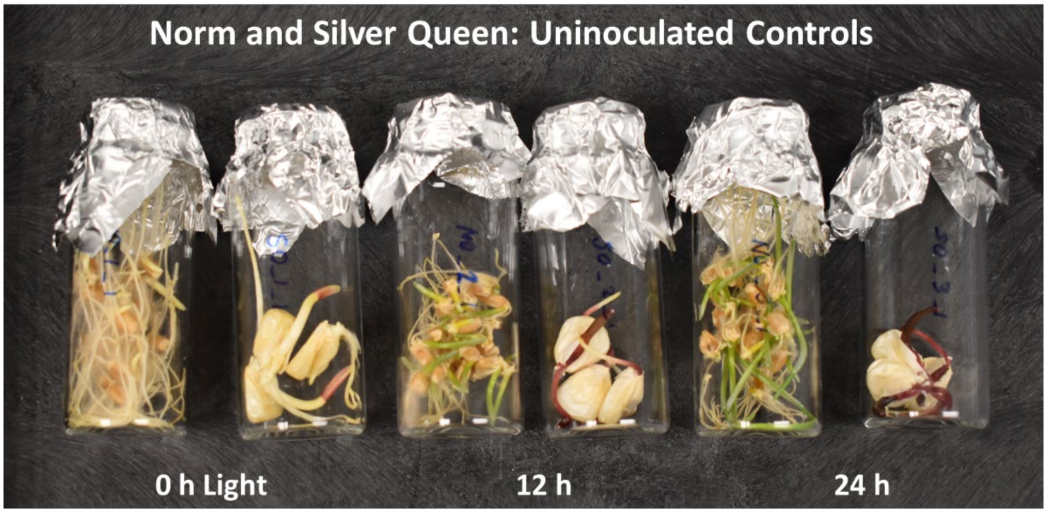

Figure S5. Wheat (Norm) and Corn (Silver Queen) controls - surface sterilized and uninoculated. One week growth in 0, 12, or 24 h/day fluorescent light (60 μmol/m^{-2} sec) at 25 °C.

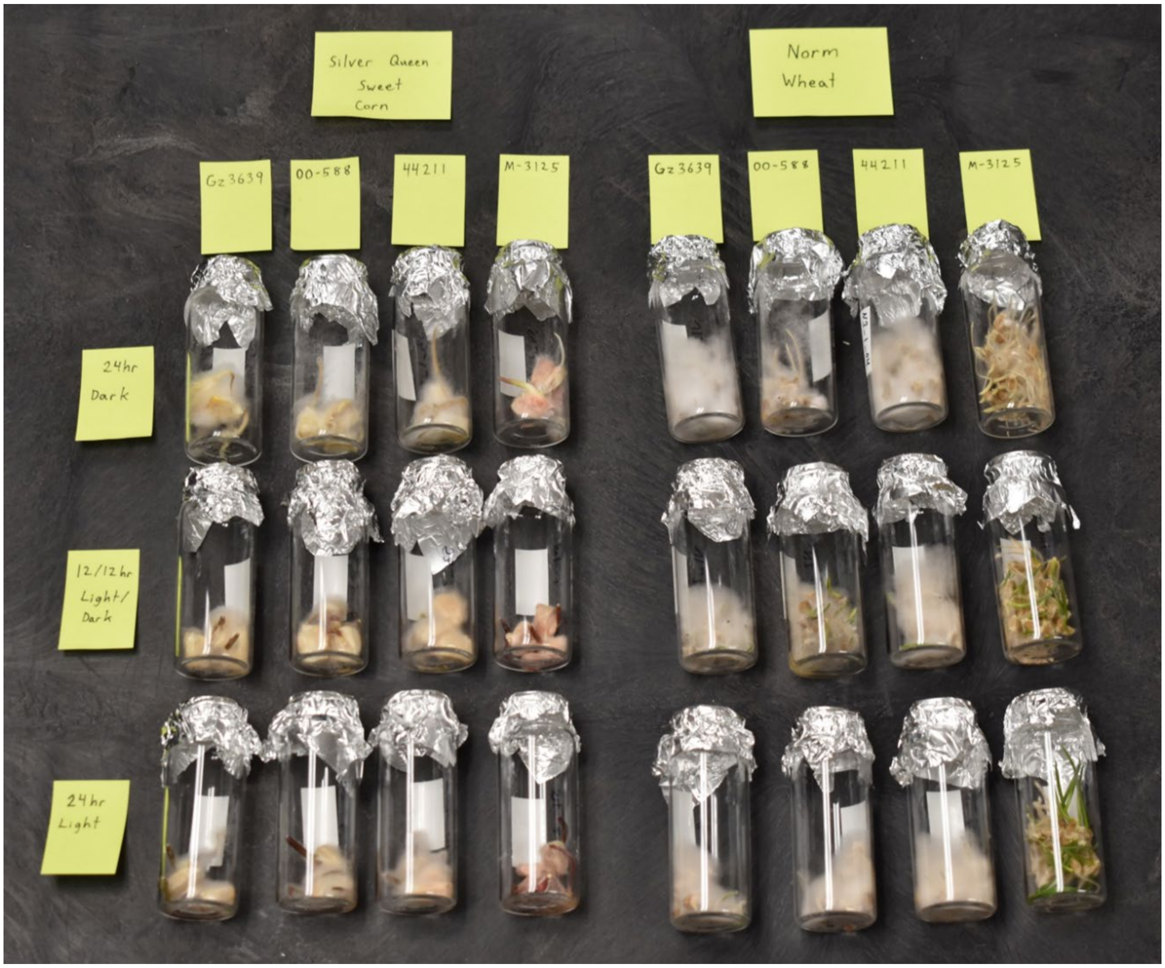

Figure S6. Representative samples of *Fusarium* inoculated corn and wheat after one week at 0, 12, or 24 h/day of fluorescent light (60 µmol/m^{-2} sec) at 25 °C. *Fusarium* strains Gz3639 (NA1), 00-588 (NA2), 44211 (NA3). Fusarium *verticillioides* (M-3125).
